# Sensory and Physicochemical Evaluation of Dairy‐Like Plant‐Based Milk Formulations Predicted by Machine Learning

**DOI:** 10.1111/1750-3841.71104

**Published:** 2026-05-10

**Authors:** Anesu A. Magwere, Russell Keast, Joanna M. Gambetta, Nelum Pematilleke, Sonja Kukuljan, Amy Logan

**Affiliations:** ^1^ Deakin Centre for Advanced Food Sciences, School of Exercise and Nutrition Sciences Deakin University Melbourne Australia; ^2^ CSIRO, Food Innovation Centre Werribee VIC Australia; ^3^ School of Environmental and Life Sciences, College of Engineering, Science and Environment University of Newcastle Ourimbah NSW Australia; ^4^ Noumi Limited Ingleburn NSW Australia

## Abstract

Plant‐based milk alternatives (PBMAs) are now increasingly used by some consumers in similar ways to cow milk. However, they differ in their physicochemical and sensory properties. Improving PBMAs to better mimic dairy is limited by the time and cost of development trials. Machine learning offers a promising approach to predict optimal formulations without the need for extensive trial and error. In this study, Random Forest regression (RFR) and gradient boosting regression (GBR) models were used to formulate PBMAs (Formulation 1 and 2, respectively) that mimic cow milk. Sensory analysis, viscosity, stability, color, particle size, and headspace volatiles were compared to cow milk to assess model accuracy. Both formulations were sensorially distinct from coconut, oat, soy, almond, pasteurized, and ultra‐high temperature (UHT) cow milk, associated with vanilla, caramel, astringent, bitter, metallic, and nutty notes, compared to the sweet, dairy, and coconut notes of dairy. The formulations contained aldehydes, including hexanal and pentanal from soy, although compounds including vanillin, octanal, and 1‐pentanol were shared with cow milk. UHT cow (0.68 µm) had the smallest average particle size (*D*_50_), followed by Formulation 2 and pasteurized cow (1.04 µm), with Formulation 1 (1.62 µm) as the largest. The UHT and pasteurized cow milk had higher whiteness indices compared with Formulation 1 and Formulation 2. Formulations exhibited faster sedimentation compared to cow milk. The models correctly predicted viscosity and some volatiles. However, predictive performance was limited by the small dataset and restricted access to commercial formulations. A larger dataset and access to formulations would improve model accuracy.

**Practical Applications:**

This research demonstrates the potential of predictive modelling for the development of plant‐based alternatives to animal‐derived ingredients. Such an approach would reduce reliance on trial and error in product development, saving time and development costs while meeting consumer needs.

## 1 Introduction

The consumption of plant‐based milk alternatives (PBMAs) products is expected to increase, with Australia's PBMA market currently valued at US$396 million and projected to grow at a compound annual growth rate of 10.34% (Mordor Intelligence 2024). This increase is attributed to environmental concerns, ethical issues related to animal welfare, and health considerations such as lactose intolerance and dairy allergies, or perceptions that PBMAs are healthier than dairy (Aydar et al. 2020; Cardello et al. 2022; Phan et al. 2026). Cow milk provides higher nutritional value than PBMAs (Sterup Moore et al. 2024), and achieving a comparable nutritional equivalence in PBMAs represents a substantial scientific challenge. Nonetheless, some PBMAs have been adopted for applications similar to cow milk, such as coffee creamer (Gupta et al. 2025). This popularity is strongly associated with taste preferences, with 39% of surveyed consumers citing better taste as a key factor for repeat purchasing behavior (Focus Insights 2024).

Dairy milk is characterized by its creamy white appearance, slight sweetness, and bland flavor (Pingali et al. 2023; Alvarez 2008). The sensory characteristics of PBMAs, on the other hand, are largely determined by their raw ingredients, with some imparting off‐flavors (e.g., beany, earthy, and grassy), astringency, bitterness, and undesirable textural attributes, including chalkiness (Lee et al. 2024; Molina et al. 2025). This difference creates a significant gap that hampers consumer adoption. Studies confirm that those who desire dairy‐like attributes, such as creaminess, whiteness, and flavor prefer PBMAs that mimic the milky flavor, aftertaste, smoothness, and mouth coating texture of dairy milk (Amyoony et al. 2023; Jaeger et al. 2024).

To the authors’ knowledge, no commercially available PBMA fully replicates the sensory and physicochemical profile of cow milk, highlighting a market gap and pointing to the complexity, cost, and time‐intensive nature of achieving product parity (Kim and Wilemon 2003). Replicating the sensory attributes of dairy milk in PMBAs is challenging, as it depends on multiple physicochemical factors, including chemical composition, emulsion stability, and viscosity (Magwere et al. 2025a), which would require an optimal balance of plant‐based ingredients to produce a successful product. Consequently, the development of PBMAs exemplifies a resource‐intensive, multi‐step new product development process, encompassing idea generation, concept and market testing, product and marketing strategy development, business analysis, and commercialization (Rudder et al. 2001). Moreover, this process can take up to five years, with 50–75% of products ultimately failing (Omniblend 2021; Dijksterhuis 2016).

Artificial intelligence (AI) has been increasingly integrated into the food industry, driving innovations in quality control, nutritional analysis, and traceability (Yang et al. 2025; Ribeiro et al. 2021; Rossi et al. 2025). Beyond this, regression‐based machine learning models, such as Random Forest regression (RFR) and gradient boosting regression (GBR), show strong potential for predictive modeling in product formulation and development. Machine learning models are mathematical representation trained on data to learn relationships between input variables and output responses, enabling prediction for unseen data (Zafar et al. 2024). RFR combines predictions from multiple decision trees to produce a single aggregated output, whereas GBR sequentially combines weak learners (simple models trained to correct previous errors) to progressively generate a stronger overall prediction (Nadkarni et al. 2023). Both RFR and GBR demonstrate strong performance when handling high‐dimensional, large and non‐linear datasets without significant over fitting (Breiman 2001; Andrade Cruz et al. 2022; AIML.com 2025; Khan et al. 2024; Airlangga and Liu 2025). Their ability to capture non‐linear interactions among physicochemical, ingredient and quality variables allows the discovery of complex relationships, often difficult to achieve through trial and error. This capability enables more accurate and efficient formulation strategies for improved physicochemical and sensory performance.

This study employed instrumental analyses to evaluate the predictive performance of RFR and GBR models in developing plant‐based milk formulations (Formulation 1 and 2, respectively) designed to closely mimic the physicochemical properties of cow milk and to assess whether these formulations also achieved sensory similarity to dairy. We believe this approach to product development may allow the agrifood industry to meet the growing demand for high‐quality PBMAs that closely replicate dairy milk while minimizing development time and cost.

## 2 Materials and Methods

### 2.1 Materials

Four commercial ultra‐high temperature (UHT) PBMAs (oat, soy, coconut, and almond milk), one commercial UHT full cream cow milk (“UHT cow milk”), and one commercial pasteurized full cream cow milk (“pasteurized milk”) were purchased locally in Melbourne, Australia. Multiple cartons (∼10) were purchased for each sample from the same batch number. With the exception of the pasteurized milk, all samples were of the same brand and composition reported in an earlier study (Magwere et al. 2025a). UHT samples were stored at room temperature (20°C), and the pasteurized cow milk was kept refrigerated at 4°C. Ingredients for formulated milks were provided by Noumi Ltd, Biospringer, Omya Invita NZ, and Alexander Ingredients. Other additional ingredients were purchased from local markets. The composition of the commercial and formulated milk samples is shown in Suppl. Table 1. The reference standards used for sensory analysis were also purchased locally and are listed in Suppl. Table 2.

Reagents used for headspace solid phase micro extraction gas chromatography mass spectrometry (HS‐SPME GC‐MS) were purchased from CDN Isotopes Inc. (Quebec, Canada) and Sigma Aldrich (Victoria, Australia). The reagents used were d_13_‐hexanol (98.5% purity), d_5_‐2‐hexanone‐1.1.3.3 (98.9% purity), d_6_‐2‐methylpyrazine (99.8% purity), d_12_‐hexanal (98.5% purity), d_11_‐3‐methylbutanol (98.9% purity), and methanol (99.8% purity).

### 2.2 Methodology

#### 2.2.1 Formulation Preparation

The two model predicted formulations (referred to herein as “Formulation 1” and “Formulation 2”) were prepared in a food‐grade pilot plant at CSIRO's Food Innovation Centre in Melbourne, Australia. The formulation process is outlined below:

##### 2.2.1.1 Data Preprocessing, Model Training and Formulation Generation

All data preprocessing, model development, hyper parameter tuning (the process of finding the best settings for a machine‐learning model before it learns from data) (Elgeldawi et al. 2021), and performance evaluation were implemented in Python (v3.12.12) using scikit‐learn (v1.6.1), with pandas and NumPy supporting data handling and numerical operations. All data were normalized, and due to the absence of commercial formulation‐level data, ingredient presence was encoded using one‐hot encoding, where the presence of an ingredient was coded as 1 and its absence as 0, to enable effective model training (Samuels 2024).

Initially, the RFR and GBR models were trained on physicochemical data as inputs (chemical composition, pH, total stability index, viscosity, whiteness index (WI), and volatile profiles) and ingredient lists to predict key physicochemical predictors in mimicking cow milk as outputs. A total of 54 commercial milk samples were analyzed and these included rice (3), a plant‐hybrid (PBMA blend), almond (10), oat (10), soy (10), coconut (10), and cow milk (10). The number of samples (*n* = 54) was constrained by the commercial availability of the products, which the authors acknowledge as a limitation in the development of the predictive model. The collated dataset (Suppl. Tables 3–7) was split into training and testing sets at an 80:20 ratio, respectively, using stratified sampling by formulation category (PBMA and cow milk) to ensure balanced representation of each milk group in both sets (Balraadjsing et al. 2024). Both models were subjected to hyper parameter tuning using randomized search with cross‐validation (RandomizedSearchCV), in which ten randomly selected hyper parameter combinations were evaluated from a predefined parameter grid using five‐fold cross‐validation (Abriha et al. 2023). The RFR and GBR models were trained based on the selected parameter in Suppl. Tables 8 and Table 9, respectively. The model performance for both RFR and GBR models was assessed based on root mean square error (RMSE) and the (coefficient of determination) *R* ^2^, which is presented in Suppl. Fig 1. Statistical differences in model performance were assessed using the non‐parametric Wilcoxon signed‐rank test (Suppl. Table 10). Thereafter, these trained models were integrated into a genetic algorithm framework, which generated ingredient types and quantities (candidate formulations) aimed at replicating cow milk physicochemical properties.

##### 2.2.1.2 Formulation Production

For each formulation, one batch (10 L) of milk was produced. Prior to production, soy protein isolate was hydrated with water at a ratio of 1:8 (w/v) and mixed at 65°C for 1 h using a heated blender (Tefal—cuisine companion) operating at speed 4. The heated soy protein mixture was transferred to 1 L plastic food containers and refrigerated overnight for use the following day. On production day, the rehydrated soy protein mixture was heated to 65°C, and all other ingredients were incorporated in a step‐wise manner according to the ratios outlined in Suppl. Table 1, with each ingredient allowed to mix for 5 min before the next was added. The resulting mixture was subjected to UHT treatment at 140°C for 4 s, followed by homogenization at 250/50 bar. The product was immediately cooled, packed into 1 L aseptic filler pouches, and stored at 5°C in the CSIRO food grade sensory kitchen until further analysis or evaluation.

#### 2.2.2 Chemical Composition

The chemical compositions of the store‐bought plant‐based and cow milk samples were obtained from the product packaging labels. The chemical composition of Formulation 1 and Formulation 2 was calculated using the Nutrition Information Panel (NIP) calculator, available on the Food Standards Australia New Zealand (FSANZ) website (https://www.foodstandards.gov.au/).

#### 2.2.3 Particle Size Distribution

The particle size distribution of the samples was analyzed according to Bernat et al. (2015), with modifications. The analysis was done using a Malvern Mastersizer 3000 (Malvern Instruments Ltd., Worcestershire, UK). A handheld refractometer (Atago Co., Ltd., Tokyo, Japan) was used to measure the refraction of each sample, and the corresponding refractive index was calculated, ranging between 1.34–1.35. Samples were added drop wise into Milli‐Q grade water (refractive index 1.33) under continuous stirring at 2500 rpm until an obscuration level between 10–12% was achieved. The *D*_50_ values were calculated using the Mastersizer 3000 software (version 3.88).

#### 2.2.4 Viscosity

A digital viscometer (DV‐II+ Version 2.0, Brookfield Engineering Laboratories, Stoughton, USA) was fitted with Spindle 00 and operated at a speed of 60 rpm to determine sample viscosity at 5°C. Aliquots (20 mL) were transferred into a custom 30 mL sample holder for measurement.

Viscosity readings were obtained in centipoise (cP) and expressed in millipascal seconds (mPa·s), using the conversion factor 1 cP = 1 mPa·s.

#### 2.2.5 Color

A Minolta colorimeter (Chromameter CR‐400, Konica Minolta Sensing, Tokyo, Japan) was used to determine sample color. Before and in‐between measurements, the instrument was calibrated with a standard white tile. Sample aliquots (15 mL) were placed in 90×15 mm Petri dishes and positioned on the colorimeter sample holder for measurement. One reading was recorded, representing the average of three measurements taken at different points on the sample surface. The color values were expressed according to the Hunter values, where *L* indicated lightness (0 = black, 100 = white), *a* denoted redness (+) or greenness (−), and *b* represented yellowness (+) or blueness (−) (McClements 2020). The overall whiteness was calculated as the WI using the equation below:

$$\begin{array}{ccc} \mathrm{Whiteness}\;\mathrm{index}\;(\mathrm{WI}) & = & 100-\sqrt{\left({\left(100-L\right)}^{2}+a^{2}+b^{2}\right)}\\ & & \;(\mathrm{Jeske}\;\mathrm{et}\;\mathrm{al}.\;\text{2017)} \end{array}$$

#### 2.2.6 Physical Stability

Backscattering was used to assess the physical stability of the samples using a Turbiscan Classic 2 (Formulaction, Toulouse, France), following the method of Matsumiya et al. (2014) with adjustments. Samples were stored overnight at 5°C prior to analysis. Each sample was hand shaken to achieve a homogeneous distribution and transferred into a 14 cm borosilicate glass tube (threaded SVL 15 top, open bottom) to a height of 6 cm. The tube was immediately placed into the Turbiscan sample holder for scanning using a laser that moved vertically along the sample tube, measuring changes in backscattering with respect to sample height and storage time (McClements et al. 2022). Following each measurement, the sample was carefully removed to avoid agitation and returned to the refrigerator (5°C) until the next day's analysis (after 24 h). Measurements were taken on the initial day (day 0) and then over the following four days, covering the manufacturer‐recommended use‐by period.

#### 2.2.7 Volatile Compounds

The headspace volatile compounds were characterized using HS‐SPME GC‐MS, using the methodology outlined by Nawaz et al. (2021), with modifications. A total of 2 µL of internal standard solution (d_6_‐2‐methylpyrazine, 8.51 mg/L; d_13_‐hexanol, 7.28 mg/L; d_12_‐hexanal, 11.3 mg/L; d_11_‐3‐methylbutanol, 14.9 mg/L; and d_5_‐2‐hexanone‐1,1,3,3, 8.74 mg/L in methanol) was added to a 20 mL headspace vial fitted with an 18 mm screw cap (Agilent Technologies, Santa Clara, USA). Aliquots (1 mL) and 0.5 g of NaCl were added to the vials, and the volatile compounds were analyzed using a Shimadzu QP2010 SE model connected to a Shimadzu AOC‐5000 Plus auto sampler (Shimadzu Europa GmbH, Duisburg, Germany). An SPME fiber (divinylbenzene/ polydimethylsiloxane/carbon wide range) (Agilent Technologies, Santa Clara, USA) was used to extract the headspace volatiles for 30 min at 40°C. The temperature was programmed to increase from 40°C to 100°C at 5°C/min, then from 100°C to 250°C at 10°C/min, and to be held at 250°C for 10 min. Desorption was carried out at 250°C, with injection in split less mode for 1 min, using helium as the carrier gas at a constant flow rate of 1.56 mL/min. Volatiles were separated on a DB‐WAX column (length 30 m, diameter 0.25 mm, and thickness 0.25 µm; Agilent Technologies).

Mass spectra were recorded using electron ionization, scanning within an m/z range of 35–350 at 0.3 s intervals. The ion source and injector temperatures were maintained at 230°C and 250°C, respectively. Compound identification was performed through comparison with the NIST Library (U.S. Department of Commerce, Gaithersburg, MD, USA). Semi‐quantitative analysis was carried out using internal standards representing similar chemical classes (Suppl. Table 11), with data processing conducted in LabSolutions software version 4.53SP1 (Shimadzu Europa GmbH, Duisburg, Germany).

#### 2.2.8 Descriptive Sensory Analysis

Ethics approval was obtained from Deakin University (2025/HE001088) and CSIRO (2021_050_LR_AJ), and informed consent was obtained from each assessor prior to their participation in the study. The evaluation was conducted according to the methodology outlined by Magwere et al. (2025a). A total of 10 trained assessors (nine females and one male aged between 25 and 55 years) were recruited from CSIRO's sensory database to participate in this study. All had prior experience (with a minimum of 40 h training and 16 h evaluations per assessor) from previous descriptive analyses of cow milk and PBMAs conducted by CSIRO. For this study, the assessors completed two 2 h training sessions within one week, followed by a third session conducted the following week, a day prior to the formal evaluation. During training, the assessors developed 29 sensory attributes and were trained to identify and rate the intensity of each attribute using reference standards (Suppl. Table 2). The final training session included practice scaling performed in individual sensory booths under 75% white light illumination, consistent with the conditions used for formal evaluation. The formal evaluations were carried out over two days, with each session lasting 2 h. In each session, assessors evaluated eight milk samples, with each milk type assessed twice across the two‐day formal testing period. Approximately 40 mL of each sample was served chilled (refrigerated at 4°C) in three‐digit coded 150 mL clear glasses and presented in a randomized order. The assessors evaluated appearance, aroma, taste, flavor, mouth feel, and aftertaste using a 0–100 structured continuous intensity scale anchored at 5 (“low”) and 95 (“high”). To prevent carryover effects, palate cleansers (plain water crackers and warm water) were provided, and a mandatory 2 min break was enforced between samples. Also, a minimum 5 min break was enforced after the first four samples, during which assessors were required to leave the booths. They were permitted to expectorate samples to minimize calorie intake. Sensory data were recorded using RedJade software (2025, RedJade Sensory Solutions LLC, CA, USA.).

#### 2.2.9 Statistical Analyses

Each instrumental analysis used four independent samples, with duplicate measurements for each sample (eight measurements in total). Sensory evaluation was conducted in duplicate for each milk type. All results were presented as the mean ± standard deviation. Statistical analyses were conducted using XLSTAT software (v2024.4.0.1424 Lumivero, CO, USA). A one‐way analysis of variance (ANOVA) was used to compare mean values across samples from instrumental analyses, and significant differences were determined using Tukey's post hoc test at a 5% significance level. For sensory analysis data, a two‐way ANOVA was used, using SPSS (IBM SPSS Statistics 30.0.0.0 (171), NY, USA), testing for assessor, sample, and presentation replicate effects and their interactions, treating assessors as a random effect.

Sensory data were further examined through principal component analysis (PCA), with non‐significant variables excluded. To determine the relationships among variables, sensory data, viscosity, WI, volatile compounds, particle size, and chemical composition were analyzed using multiple factor analysis (MFA). Both PCA and MFA analyses were conducted using XLSTAT software (v2024.4.0.1424, Lumivero, CO, USA). All data were standardized prior to performing the PCA and MFA analyses. Data visualizations of the volatile compound bubble plot were generated in Python (version 3.12.12) using Matplotlib within Google Colaboratory (Google LLC, CA, USA).

## 3 Results and Discussion

### 3.1 Chemical Composition

The chemical composition of cow milk, Formulations 1 and 2 are shown in Table 1. Formulation 1 and Formulation 2 were lower in protein compared to pasteurized and UHT cow milk samples. This is because the model‐predicted protein content was based on functional equivalence rather than nutritive equivalence, as increasing protein levels to match those of cow milk would adversely affect sensory properties. In particular, the degradation of soy protein isolate can release bitter peptides containing leucine, phenylalanine, isoleucine, and proline, which may enhance perceived bitterness in the final product (Bi et al. 2025; Bertelsen et al. 2018).

**TABLE 1:** Chemical composition of Formulation 1, Formulation 2, pasteurized, and ultra‐high temperature (UHT) cow milk types.

	g/100 g				mg/100 g	
Milk type	Protein	Total fat	Carbohydrates	Sugars	Sodium	Calcium
Formulation 1	1.4	3.4	3.6	1.2	47	20*
Formulation 2	1.4	3.4	6.0	2.4	51	20*
Pasteurized cow	3.2	3.4	4.5	4.5	43	118
UHT cow	3.3	3.5	5.1	5.1	40	117

*Note*: ^*^Calcium values for all plant‐based milk formulations were estimated using supplier‐provided compositional data from Omya (Omya International AG). Calcium content for the pasteurized and UHT cow milk samples was obtained directly from the nutrition information panels on the product packaging at the time of purchase. Calculations for the formulated samples were performed using Food Standards Australia New Zealand, Nutrition Panel calculator (https://www.foodstandards.gov.au/). Carbohydrate values include sugars.

With the exception of UHT cow milk, all samples had a total fat content of 3.4%. Fat is a major driver of milk creaminess and consumer liking, and it also plays a key role in increasing the perceived whiteness of milk (Frøst and Janhøj 2007; Phillips et al. 1995), highlighting the importance of matching fat content when mimicking cow milk. However, similar fat content does not equate to similar fatty acid compositions and, consequently, the functionality linked to them. Saturated fatty acids are known to enhance creaminess by forming compact crystalline structures with higher melting points that increase viscosity and improve mouth feel compared to unsaturated fatty acids (Scott et al. 2003; Zhou et al. 2022). Cow milk fat contains approximately 70% saturated fatty acids, compared with the 11–16% found in PBMAs (Magwere et al. 2025b). Another example is that soybeans are rich in polyunsaturated fatty acids, which are prone to oxidation and may generate characteristic beany off‐flavors (Tao et al. 2022). Hence, fatty acid type and degree of unsaturation influence both oxidative susceptibility and the nature of off‐flavors produced.

Formulation 2 had the highest carbohydrate concentration, followed by UHT cow milk, pasteurized milk, and Formulation 1. The carbohydrate fraction of both UHT cow and the pasteurized cow milk consisted of the sugar lactose. However, the carbohydrate fraction of the formulations consisted of sugars from the tapioca syrup (www.glucochem.com 2025), insoluble fiber, and other plant cell wall material from the brown rice, inulin, and soybeans (Suppl. Table 1). Carbohydrates, particularly insoluble fiber, play an important role in determining the physical properties of PBMAs. These fibers can increase particle size, which may lead to a gritty mouthfeel and a higher rate of physical destabilization through sedimentation (Magwere et al. 2025a). At the same time, carbohydrates contribute to viscosity, overall mouth feel and texture, and sweetness perception (Wolinska‐Kennard et al. 2025; Kokkinidou et al. 2018). The presence of reducing sugars is also relevant during thermal processing, as they can influence the rate and extent of caramelization and Maillard reactions, which in turn affect flavor and brown color development (Różańska et al. 2025).

Sodium values ranged between 40–51 mg/100 g. Calcium content was identical in Formulations 1 and 2, while pasteurized and UHT cow milk had substantially higher concentrations (Table 1). Sodium is typically added to PBMAs as a flavor enhancer to improve overall palatability, while calcium is added for nutritional purposes and to enhance the whiteness (Vanga and Raghavan 2018; Liem et al. 2011; Younes et al. 2023). However, the behavior of calcium differs markedly between PBMAs and dairy milk. In cow milk, calcium is primarily incorporated within the casein micelle as calcium phosphate nanoclusters, contributing to its colloidal stability. In contrast, calcium in PBMAs is present as insoluble mineral particles that may agglomerate within the milk (Beckett et al. 2024). This structural difference reduces colloidal stability and promotes faster sedimentation in PBMA formulations.

### 3.2 Particle Size Distribution

The particle size distribution parameters of the milk samples are presented in Table 2 and are expressed as *D*_50_ values, representing the particle diameter below which 50% of the particle volume is distributed (Nobbmann 2025). Significant differences were observed among the samples, with Formulation 1 exhibiting the largest particle size, followed by Formulation 2 and pasteurized cow milk, and lastly UHT cow milk. The particle size distribution of Formulation 1 and Formulation 2 varied, despite undergoing similar production processes using the same ingredients and identical homogenization conditions (250/50 bar). These differences were likely due to batch variability. Despite the observed significante differences, all samples in the study exhibited particle sizes that fall within the ranges of homogenized milk reported in the literature (Van Hekken et al. 2017).

**TABLE 2:** Particle size characterization and viscosity of Formulation 1, Formulation 2, pasteurized, and ultra‐high temperature (UHT) cow milk types.

Milk type	*D*_50_ (µm)	Viscosity (mPa·s)
Formulation 1	1.51 ± 0.07^a^	1.87 ± 0.20^a^
Formulation 2	1.04 ± 0.03^b^	1.80 ± 0.26^a^
Pasteurized cow	1.04 ± 0.03^b^	1.86 ± 0.13^a^
UHT cow	0.68 ± 0.01^c^	1.86 ± 0.05^a^

*Note*: Values in the same column with different superscript letters differ significantly (*p* < 0.05). *D*_50_ represents the diameter below which 50% of the volume of particles are found.

### 3.3 Viscosity

All samples had similar viscosity measurements, with values ranging from 1.80–1.87 mPa·s (Table 2). Since milk viscosity is strongly associated with fat content (Li et al. 2018; Iqbal et al. 2024), the similar fat concentrations among samples (Table 1) could have contributed to the absence of statistical differences. Importantly, the low variability in viscosity is crucial because PBMAs are often penalized by consumers for having a thin and watery texture (Jaeger et al. 2024; Collier et al. 2023). Achieving a viscosity comparable to dairy milk is therefore a meaningful outcome, as viscosity is a major driver of perceived creaminess (Dickinson 2018) and is key to improving dairy mimicry. In addition to fat, the inclusion of xanthan gum (Suppl. Table 1) contributed to viscosity enhancement. This is because when hydrocolloids such as xanthan gum are added to water, they swell and form a gel‐like network that increases viscosity (O'Sullivan and O'Mahony 2016).

### 3.4 Color

The color parameters (*L*, *a*, *b*) and WI of the milk samples are presented in Table 3. The significantly higher *L* value of Formulation 1 compared with Formulation 2 may have been attributed to its lower sugar concentration (Table 1), where Formulation 2 contained double the amount of sugar (2.4%) than Formulation 1 (1.2%). Higher sugar levels in milk increase both the rate and the overall yield of browning reactions, including Maillard reactions and caramelization, which can darken it (El Hosry et al. 2025; Lee and Lee 1997), lowering the *L* value. Compared to UHT and pasteurized cow milk, both formulations had significantly lower *L* values. Previous research indicates that the fat and protein content play an important role in determining the degree of lightness in milk, with higher fat and protein concentrations associated with higher *L* values (Phillips et al. 1995; Quiñones et al. 1998). In the present study, however, all four samples had comparable fat contents, with the UHT cow milk sample only slightly higher (Table 1). Additionally, both olive oil (Suppl. Table 1; fat source in the formulations) and cow milk fat globules have the same refractive indices (1.46–1.47) (Douzane et al. 2021; Michalski et al. 2001), further indicating that differences in fat optical properties were minimal across these samples. Therefore, fat content and type alone could not fully explain the differences in lightness observed in this study. However, both formulations contained lower protein levels than the cow milk samples (Table 1) and lacked casein micelles, which are key contributors to light scattering (Misawa et al. 2016). This absence could have accounted for the reduced lightness observed in the formulations.

**TABLE 3:** Effect of Formulation 1, Formulation 2, pasteurized, and ultra‐high temperature (UHT) cow milk types on color.

Milk type	*L*	*a*	*b*	Whiteness index
Formulation 1	66.09 ± 0.13^b^	−0.64 ± 0.02^a^	6.32 ± 0.03^b^	65.50 ± 0.13^b^
Formulation 2	62.55 ± 3.22^c^	−1.09 ± 0.17^ab^	4.84 ± 0.56^c^	62.22 ± 3.19^c^
Pasteurized cow	72.45 ± 0.02^a^	−1.77 ± 1.40^b^	6.76 ± 0.01^a^	71.54 ± 0.02^a^
UHT cow	71.41 ± 0.71^a^	−2.75 ± 0.17^c^	3.70 ± 0.20^d^	71.04 ± 0.67^a^

*Note*: Values in the same column with different superscript letters differ significantly (*p* < 0.05). The color values were expressed according to the Hunter values, where *L* indicated lightness (0 = black, 100 = white), *a* denoted redness (+) or greenness (−), and *b* represented yellowness (+) or blueness (−) [36].

All samples displayed a relatively pronounced green hue (negative *a* values), with UHT cow milk appearing the most green, likely due to its higher concentration of green chromophores such as riboflavin (Misawa et al. 2016). Pasteurized milk showed some degree of greenness, although with a lower intensity than UHT cow milk, which was attributed to natural variations in raw milk chromophore composition. A similar explanation applies to the yellowness observed in all the samples, which is known to be caused by the presence of carotenoids (Iwuoha and Umunnakwe 1997; Conboy Stephenson et al. 2021). The concentration of these chromophores can vary depending on the animal's diet (i.e., grass silage vs hay), or seasonal changes, contributing to color differences between the cow milk samples (Agabriel et al. 2007; Poulsen et al. 2015; Solah et al. 2007). In comparison, Formulation 1 and Formulation 2 were less green than UHT cow milk, as their color is influenced by chlorophyll naturally present in plants, including soybeans (Gebregziabher et al. 2022), but could have been present at lower levels than the riboflavin.

The WI is determined by the combined contribution of *L*, *a*, and *b* values to give an overall value between 0–100 (Milovanovic et al. 2020). Formulations 1 and 2 exhibited lower whiteness compared with pasteurized and UHT cow milk. Misawa et al. (2016) reported that increased light scattering reduces the absorbance of wavelengths responsible for green and yellow appearance. This supports a positive relationship between *L* and overall whiteness. Consequently, the higher *L* values of cow milk contributed to its comparatively higher WI relative to the formulations.

### 3.5 Physical Stability

**FIGURE 1:**
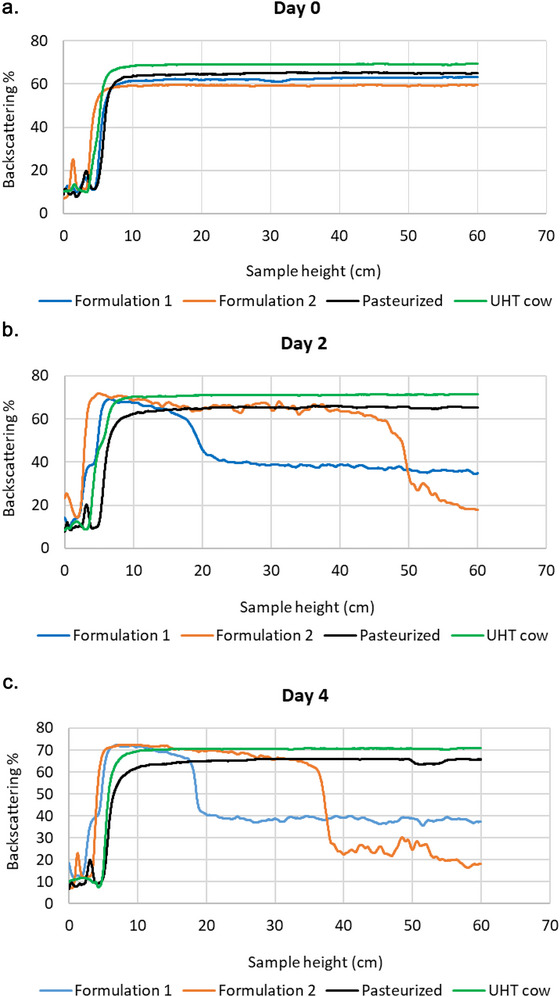
a. Backscattering intensity % versus sample height of Formulation 1, Formulation 2, pasteurized cow, and UHT cow milk types on (a) day 0, (b) day 2 and (c) day 4. Values presented as mean values of 4 repetitions (*n* = 4).

The Turbiscan was used to assess destabilization patterns in the milk samples by monitoring particle migration (creaming or sedimentation) and detecting increases in particle size changes associated with coalescence (Kowalska et al. 2020), via backscattering intensity measurements. Figures 1a, 1b, and 1c show the backscattering intensity (%) of each milk sample from day zero to day four across the 60 cm sample height. On day zero, all samples showed similar horizontal backscattering profiles, indicating homogeneous particle distribution immediately after agitation. While the overall pattern was consistent, backscattering intensities differed, with UHT (69%) and pasteurized (65%) cow milk showing the highest values, likely due to increased light scattering from casein micelles (Cheng et al. 2019). From day two, all samples demonstrated measurable instability, and three distinct regions of the scan became apparent: bottom (0–20 cm), middle (20–40 cm), and upper (40–60 cm). Formulation 1 showed increased backscattering at the bottom and middle regions by 6.51% and 23.04%, respectively, and a slight increase of 1.46% in the upper region. Formulation 2 showed a backscattering increase in the bottom (11.71%), middle (6.12%), and a 17.54% decrease in the upper region. For both cow milk samples, all three regions had minor changes < 3%. On day four, Formulation 2 showed the largest decrease in the upper region (17.54%). Changes in the bottom region remained small, with increases of 2.16% (Formulation 1), 1.66% (Formulation 2), and 0.45% (pasteurized cow milk), and a 2.44% decrease in UHT cow milk.

Both Formulation 1 and Formulation 2 exhibited an increase in backscattering intensity at the bottom of the tube, accompanied by a decrease in the middle and upper regions. The increased intensity at the bottom indicated the onset of sedimentation, while decreases in the middle and upper regions of the sample tube were due to clarification (sample separation) (Tirpanci et al. 2023). The clarification may have occurred due to gravitational separation, which led to the downward migration of insoluble plant matter particles such as proteins, mineral complexes, and fiber (McClements 2020). Formulation 1 had a faster destabilization rate relative to Formulation 2, which may be attributed to its larger *D*_50_ particle size (Table 2) and higher concentration of tapioca syrup (Suppl. Table 1). The destabilization of UHT and pasteurized cow milk was due to creaming, as the fat (due to lower density) migrated to the upper region of the tube (Magwere et al. 2025a). A ±2% variation in a colloidal system is still regarded as stable because the Turbiscan can detect extremely small changes well before any macroscopic instability becomes visible (Kowalska et al. 2020). The stability of the cow milk samples could have resulted from their stable casein micelles and homogenized milk fat globules, their particle size, which was smaller than Formulation 1 (Table 2) and the absence of denser plant‐derived material (Hu et al. 2025).

### 3.6 Volatile Compounds

**FIGURE 2:**
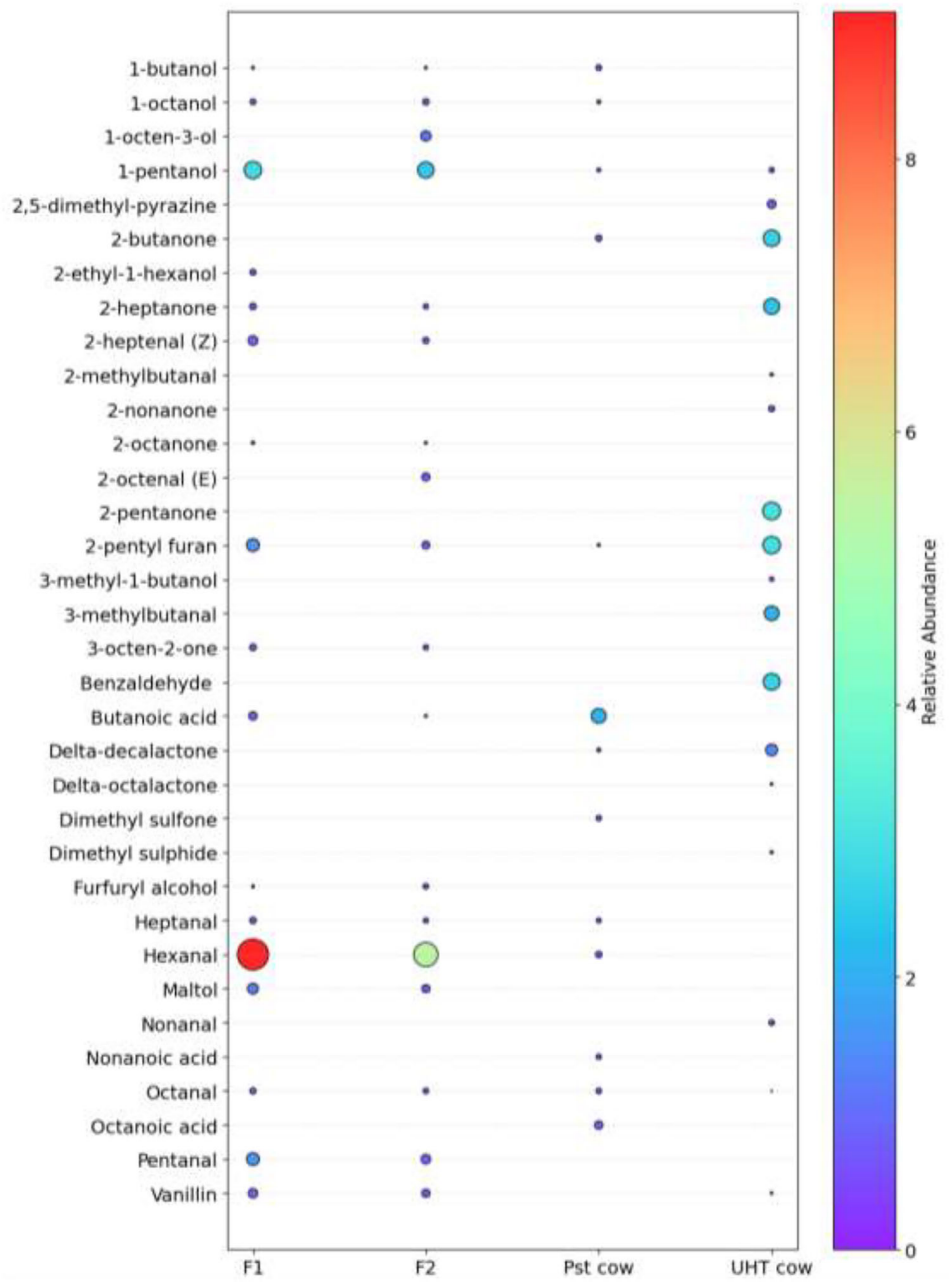
Bubble plot of the relative abundance of volatile compounds in Formulation 1, Formulation 2, pasteurized cow milk, and UHT cow milk types. In the bubble plot, each circle represents a compound, with bubble size indicating its concentration relative to other compounds within the same sample. The color gradient reflects concentration, where purple represents lower levels and red represents higher levels. F1 = Formulation 1, F2 = Formulation 2, and Pst cow = pasteurized cow.

GC‐MS analysis identified and semi‐quantified 34 volatile compounds across all samples, which were classified into major chemical groups including seven alcohols, five ketones, three acids, ten aldehydes, and others, with their relative abundances shown in Figure 2. Cow milk contained 2‐pentylfuran, which is known to form through Maillard reactions involving lactose and phenylalanine, contributing caramel notes (Li and Wang 2016). In PBMAs, 2‐pentylfuran has also been widely reported, including in studies by Feng et al. (2023), Yu et al. (2017), and McCarron et al. (2024). The volatile compound has been associated with fruity and green aromas in PBMAs (McCarron et al. 2024). Its formation in PBMAs has been attributed to two main pathways: oxidation of unsaturated fatty acids and Maillard reaction products, with the Maillard pathway generally considered the dominant route (McCarron et al. 2024; Poliseli‐Scopel et al. 2013). The formation of furans, including 2‐pentylfuran, is known to increase when milk undergoes heat treatment (Fan et al. 2008). In this study, pasteurized milk was heated at 72°C for 15 s, while all other samples were processed under UHT conditions (Jo et al. 2018). This aligns with our observation that pasteurized milk contained the least 2‐pentylfuran, while the formulations and UHT cow milk, exposed to the higher temperatures of UHT processing, exhibited higher concentrations. The difference in heat treatment conditions could also explain the variations observed in other heat‐induced compounds such as furfuryl alcohol and 2,5‐dimethyl pyrazine, which impart sweet, nutty, and chocolate aromas, respectively (Xiao et al. 2014; Pointke et al. 2022; Jiang et al. 2024). The higher furfuryl alcohol concentration in Formulation 2 than in Formulation 1 could be explained by heat‐induced Maillard reactions and its higher sugar content (Table 1), which provided more substrate for these reactions. Similarly, 2,5‐dimethyl pyrazine was present in UHT cow milk but absent in pasteurized milk, likely because the higher time–temperature conditions of UHT processing promoted its formation compared to pasteurization. Other volatiles commonly associated with UHT processing, such as benzaldehyde, 2‐pentanone, 2‐heptanone, and dimethyl sulfide, were also present in the UHT cow milk, as reported in previous studies (Jo et al. 2018; Lan et al. 2025; Contarini and Povolo 2002). These compounds impart characteristic cooked and sulfurous (benzaldehyde, 2‐heptanone, dimethyl sulfide), nutty (benzaldehyde), sweet and fruity (2‐pentanone), and waxy or green notes (2‐heptanone) (Jo et al. 2018; Lan et al. 2025). The higher temperature may have accounted for the higher levels of these compounds in UHT cow milk compared to pasteurized milk. Heat treatment in cow milk is also associated with a reduction in hexanal, as this promotes the binding of milk proteins to volatiles such as hexanal, thereby lowering the compound's detectable concentration (Lan et al. 2025). This finding explains the absence of hexanal in UHT milk, while it remained detectable in the pasteurized milk.

Since the formulations were prepared using soy protein and a soy whole‐bean premix flour (Suppl. Table 1), it was expected that their volatile profile would have some similarity to that of soymilk. According to Zhang et al. (2020) and Magwere et al. (2025a), the most dominant compounds in soy milk are aldehydes, and a similar trend was also observed in the present study. Formulations 1 and 2 contained hexanal, pentanal, and 2‐heptenal (Z), which were not detected in the cow milk types, while 2‐octenal (E) was detected only in Formulation 2. These compounds give off grassy, green (hexanal and pentanal), pungent (2‐heptenal (Z)), and fatty, and green (2‐octenal) aromas (Vaikma et al. 2021; Jiang et al. 2024; Feng et al. 2023). Both formulations also contained several alcohols, including 1‐octanol (bitter almond, fatty, green, rose), 1‐pentanol (fruity, green, grain, mushroom, vanilla), 1‐butanol (sweet), and furfuryl alcohol (sweet) (Jiang et al. 2024; Pointke et al. 2022; Zhogoleva et al. 2023). Only Formulation 2 contained 1‐octen‐3‐ol (mushroom aroma), while 2‐ethyl‐1‐hexanol (oily, floral, sweet aroma) was detected only in Formulation 1 (Vaikma et al. 2021; Jiang et al. 2024).

Both UHT and pasteurized cow milk were unique in containing lactones, specifically δ‐octalactone and δ‐decalactone, which are compounds associated with the characteristic milky aroma of dairy (Schütt and Schieberle 2017). Vanillin was detected in all samples except pasteurized cow milk. In cow milk, heat treatment can generate vanillin by oxidizing coniferyl alcohols, sourced from rumen microbial breakdown of plant lignin (Calvo and de la Hoz 1992). This differs from the vanillin found in the formulations, which is present due to the added vanilla flavoring (Suppl. Table 1). Other significant compounds found in pasteurized cow milk were heptanal (earthy, fatty), octanoic acid (acidic, waxy), dimethyl sulfone (sulfurous, burnt), and 2‐butanone (plastic) (Jo et al. 2018; Jiang et al. 2024). Octanal was also present in all four samples and is associated with fruity and fatty notes, and has also been reported in literature to be present in cow milk and PBMAs (Faulkner et al. 2018; Achouri et al. 2008; Feng et al. 2023). Overall, the formulations shared some key volatiles with cow milk, indicating partial mimicry of dairy aroma.

### 3.7 Descriptive Sensory Analysis

Physicochemical analyses were conducted on four samples (Formulation 1, Formulation 2, UHT milk, and pasteurized milk), whereas descriptive sensory analysis was performed on eight samples to allow clearer sensory mapping of the formulations relative to commercial PBMAs. The physicochemical properties of the additional samples included in the sensory analysis were previously characterized in Magwere et al. (2025a).

**FIGURE 3:**
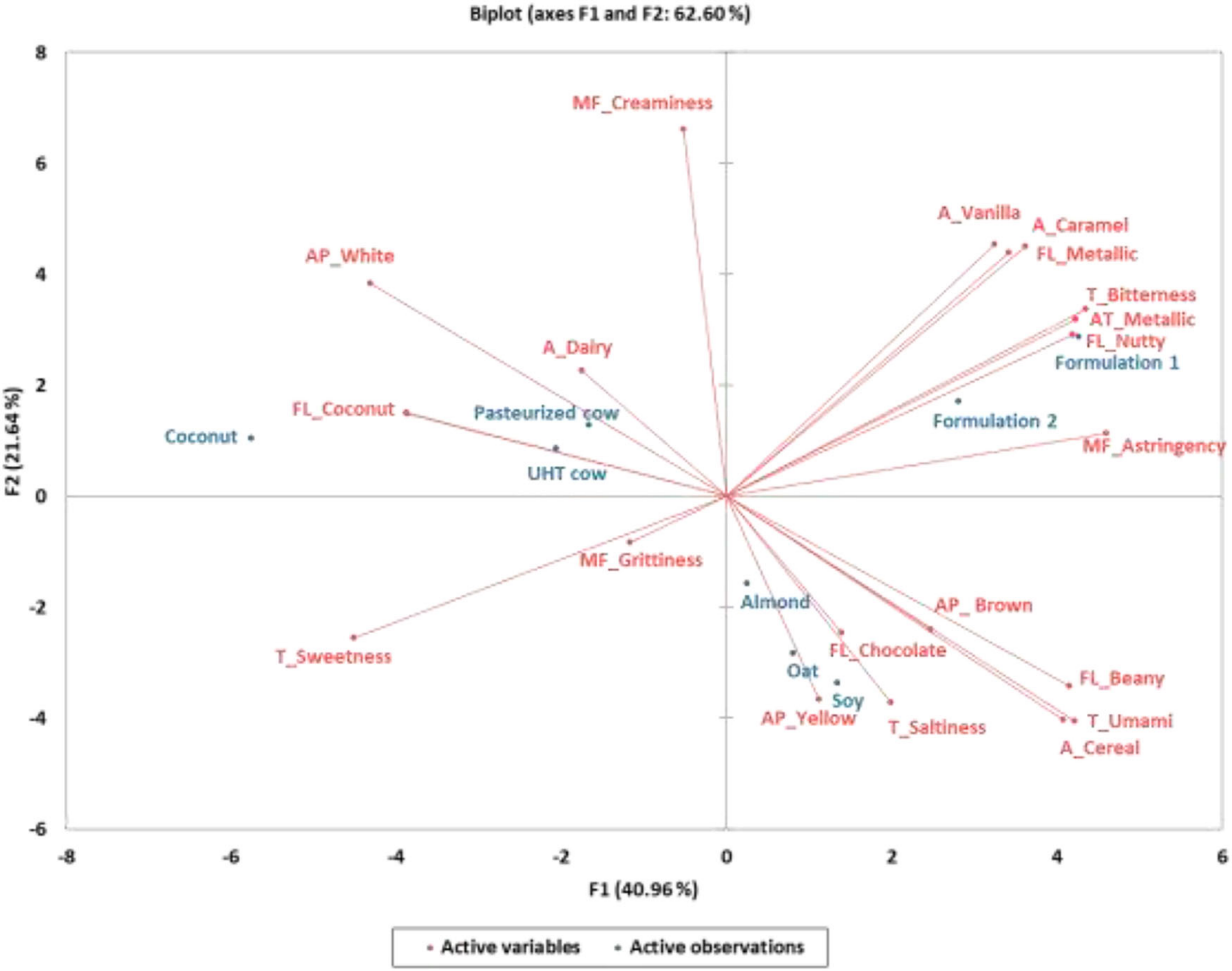
Principal component analysis of sensory descriptive analysis of pasteurized cow (pasteurized cow), Ultra high‐temperature cow (UHT cow), almond, coconut, oat, soy, Formulation 1 and Formulation 2 milk samples. Sensory attribute prefixes: AP_ appearance, A_ aroma, T_ taste, FL_ flavor, and MF_ mouthfeel.

The assessors evaluated 29 sensory attributes, among which only sourness was found to be non‐significant (Suppl. Table 12), and PCA was conducted on the remaining significant attributes. Factors 1 and 2 (Figure 3) explained 62.6% of the total variance, with Factor 1 (F1) accounting for 40.96% of the variance and Factor 2 (F2) of 21.64%.

The PCA biplot showed separation of milk samples, with coconut, pasteurized cow milk, and UHT cow milk clustering in Quadrant 1, driven mainly by white color intensity and creaminess, with contributions from dairy aroma and coconut flavor. Both pasteurized and UHT cow milk samples clustered within the same sensory quadrant, which is likely attributable to their shared animal origin, despite differences in thermal processing. These samples, along with coconut milk, were associated with creaminess, dairy aroma, and whiteness, reflecting the multifactorial nature of creaminess. Creaminess is considered an integrated sensory construct comprising both flavor and texture components, including dairy flavor, smoothness, and thickness (Chen and Eaton 2012; Dickinson 2018). In addition, Quiñones et al. (1997) reported that in skim and 1% fat cow milk, greater whiteness increased assessors’ perception of thickness. In this context, whiteness could have acted as a visual cue associated with thickness and therefore contributed indirectly to the perception of creaminess. Coconut milk was also grouped with cow milk samples, likely due to its naturally high lactone content (28.74%), which is known to impart a creamy, dairy‐like flavor (Xing et al. 2025; Schlutt et al. 2007). This was evident from the presence of γ‐decalactone, δ‐octalactone, γ‐octalactone, and δ‐undecalactone in the sample (Magwere et al. 2025a). Additionally, its high coconut cream level (15%) (Suppl. Table 1) provided substantial fat, which may have enhanced perceived creaminess and increased whiteness (Phillips et al. 1995; Chi et al. 2025), further aligning its sensory profile with cow milk. This is consistent with the WI values (Table 3), as the cow milk samples were the whitest, and coconut milk was deemed even whiter than cow milk in the analysis by Magwere et al. (2025a). The importance of color attributes (yellow and brown) was further confirmed by the Factor 3 (F3) component, where cow milk samples were grouped together, indicating similar contributions of these attributes. This component also reflected sample classification based on grittiness and chocolate flavor.

Formulation 1 and Formulation 2 were positioned in Quadrant 2 and were associated with vanilla aroma, caramel aroma, metallic flavor and aftertaste, bitterness, nutty flavor and astringency, likely due to their similar ingredient compositions, as shown in Suppl. Table 1. Vanilla and caramel aromas resulted from added flavorings, with vanilla extract commonly used in PBMAs to enhance flavor and mask undesirable notes, thereby improving consumer acceptability (Manzoor et al. 2017; Rincon et al. 2020). However, the authors found no evidence in the literature that reports the use of caramel flavoring in PBMAs. This suggests that the models included these flavorings to mimic the vanilla and caramel notes of UHT cow milk, which are linked to vanillin (detected in the samples; Figure 2) and heat treatment, respectively (Zhu and Xiao 2017; Jiang et al. 2024; Jo et al. 2018). This rationale may also account for the addition of maltol to replicate the natural sweetness of cow milk (Zhu and Xiao 2017).

Both Formulations 1 and 2 contained a soy base (2% soy protein and 2% whole soybean premix), which may explain the bitter, metallic, and astringent attributes observed. These sensory characteristics have been consistently reported as inherent attributes of soy milk in previous studies (Torres‐Penaranda and Reitmeier 2001; Vaikma et al. 2021). Quadrant 3 contained almond, oat, and soy samples, which were mainly associated with umami, cereal aroma and beany flavor. These samples were also linked to yellow and brown colors, chocolate flavor, and saltiness. Almond milk has been associated with chocolate notes and a brown hue/characteristic white, oat milk with a cereal aroma, and soy milk with a yellow/off‐white coloration (Magwere et al. 2025a; Iwuoha and Umunnakwe 1997; Achouri et al. 2008; Alozie et al. 2015). The yellowness in soy milk is due to chromophores naturally present in soybeans, including carotenoids (Iwuoha and Umunnakwe 1997; Conboy Stephenson et al. 2021). The brown color observed in almond milk arises from the roasting of almonds before processing, which induces Maillard browning reactions (Franklin and Mitchell 2019). Despite similarities in viscosity and the presence of certain volatile compounds, neither formulation successfully replicated the sensory profile of dairy. This highlighted that sensory perception does not always correlate directly with individual physicochemical attributes, as it is shaped by multiple interacting factors. Consequently, physicochemical measurements provided complementary rather than definitive insight into consumer‐perceived quality (Saleh and Lee 2023). Furthermore, as outlined in Section 3.1, fundamental differences in chemical structure and composition, including the presence of lactose in dairy compared with complex carbohydrates in plant‐based formulations, variations in fatty acid profiles, and inherent ingredient characteristics such as protein‐associated bitterness and astringency, likely constrained the extent to which complete sensory equivalence with dairy could be achieved.

### 3.8 Multiple Factor Analysis

**FIGURE 4:**
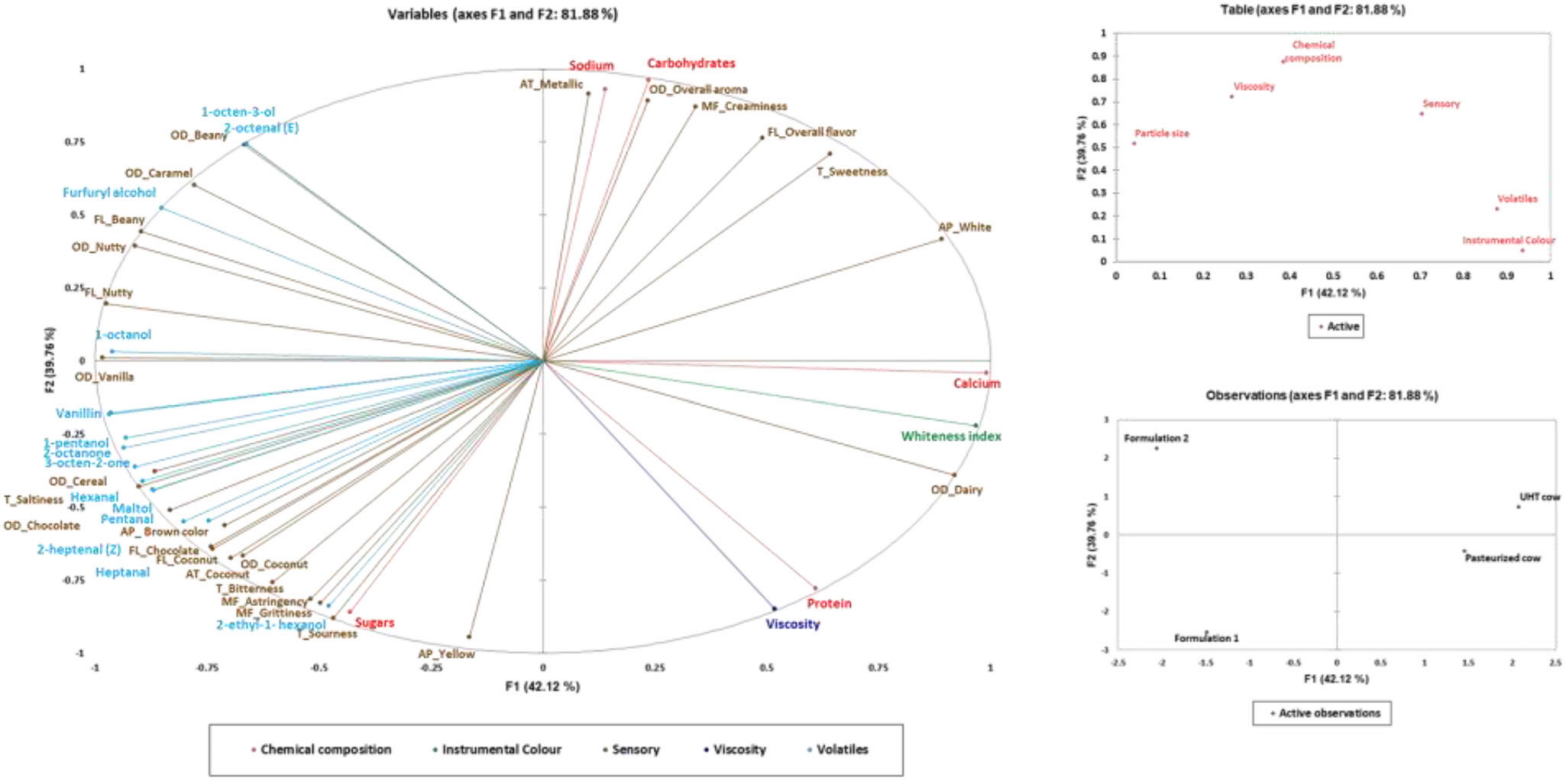
Multifactor analysis correlating chemical composition, color, particle size, sensory attributes, viscosity, and volatile compounds. Sensory attribute prefixes: Ap_ indicates appearance, OD_ indicates aroma, T_ indicates taste, FL_ indicates flavor, and MF_ indicates mouthfeel.

MFA was conducted to integrate sensory, volatile, instrumental color (WI), viscosity, particle size, and chemical composition datasets and to identify the major dimensions describing variation across the samples (Figure 4). The first dimension (F1) accounted for 42.12% of the total variability, while the second dimension (F2) explained 39.76%, giving a cumulative explained variance of 81.88%.

The MFA revealed very strong associations between the sensory and chemical composition data (RV coefficient = 0.90). Examination of variable representation showed that viscosity and protein content were strongly associated with F2 (cos^2^ = 0.72 and 0.61, respectively). The WI clustered with calcium content (cos^2^ = 0.98) and dairy aroma attributes (cos^2^ = 0.85) on F1. Carbohydrates (cos^2^ = 0.93) were associated with overall aroma intensity (cos^2^ = 0.80), creaminess (cos^2^ = 0.76), and white appearance (cos^2^ = 0.80). Sugar content (cos^2^ = 0.74) was also associated with brown color intensity (cos^2^ = 0.90).

Nutty, beany, and chocolate attributes were strongly represented on F1 (cos^2^ = 0.95, 0.61, and 0.69, respectively), whereas grassy/green‐related volatiles, including hexanal (cos^2^ = 0.80), 1‐hexanol‐2‐ethyl (cos^2^ = 0.70), and 1‐octen‐3‐ol (cos^2^ = 0.55), were associated with F2 (Feng et al. 2023; Jo et al. 2018; Vaikma et al. 2021). Protein content (cos^2^ = 0.61) was correlated with viscosity (cos^2^ = 0.72).

The squared cosines of the observations indicated that the pasteurized and UHT cow milk samples were mainly represented on F1 (cos^2^ = 0.39 and 0.63, respectively), whereas Formulation 1 and Formulation 2 were primarily represented on F2 (cos^2^ = 0.72 and 0.54, respectively). The scores plot showed that F1 was predominantly associated with instrumental color (cos^2^ = 0.88), volatiles (cos^2^ = 0.56), and sensory data (cos^2^ = 0.42), while F2 was mainly associated with chemical composition (cos^2^ = 0.67), viscosity (cos^2^ = 0.52), and particle size (cos^2^ = 0.27).

## 4 Conclusion

The RFR and GBR AI‐predicted formulations successfully matched cow milk for viscosity and shared some overlapping volatile compounds such as octanal, 1‐pentanol, and vanillin, indicating meaningful progress toward dairy mimicry. However, notable differences remained in whiteness, physical stability, and key aroma compounds such as lactones, which prevented full replication of the dairy sensory profile. Moreover, the sensory analysis confirmed that the formulations were positioned closer to PBMAs than to cow milk, and that further work is needed through potential broadening of the dataset used to test each model and bridge the gap. The current models were limited by the unavailability of commercial formulations and a small dataset. However, despite the obvious differences, the ingredient blends used herein suggest a shift away from the defining attributes of any single dominant PBMA, potentially as a strategy to minimize undesirable attributes while achieving a more balanced overall profile. The integrated MFA further highlighted the combined influence of composition, volatiles, color, and structural attributes on the overall perception of dairy mimicry.

Overall, we have shown AI‐driven modeling proved valuable in narrowing ingredient combinations, with potential to reduce formulation time after iterative improvements to each model are employed. Future studies should place greater emphasis on expanding model inputs, including optimization of protein structure, mineral incorporation, flavor generation, and colloidal stability. This work highlights the potential of predictive modeling as a powerful tool in accelerating PBMA innovation and provides a foundation for future refinement toward.

## Author Contributions

**Anesu A. Magwere**: conceptualization, methodology, data curation, investigation, validation, formal analysis, visualization, writing – original draft, writing – review and editing. **Russell Keast**: conceptualization, methodology, supervision, writing – review and editing. **Joanna M. Gambetta**: conceptualization, methodology, writing – review and editing. **Nelum Pematilleke**: methodology, writing – review and editing. **Sonja Kukuljan**: conceptualization, methodology, writing – review and editing. **Amy Logan**: conceptualization, methodology, writing – review and editing, supervision.

## Conflicts of Interest

The authors declare no conflicts of interest.

## Supporting information




**Supplementary Material**: jfds71104‐sup‐0001‐SuppMat.docx

## Data Availability

The data that support the findings of this study are available from the corresponding author upon reasonable request.
