# Supplementary material for: Sensory and Physicochemical Evaluation of Dairy‐Like Plant‐Based Milk Formulations Predicted by Machine Learning
Source: J Food Sci. 2026 May 10;91:e71104. doi: 10.1111/1750-3841.71104 (PMC13158331; doi:10.1111/1750-3841.71104)
Supplement: Supplementary file 1 — Supplementary Material: jfds71104‐sup‐0001‐SuppMat.docx [file JFDS-91-0-s001.docx]

**Supplementary Table 1: Ingredient list for (a) the oat, soy, almond, coconut, pasteurized cow, and UHT cow milk types, as per manufacturers' declaration, and (b) the experimentally formulated milks**

| **Milk type** | **Ingredients** |
| --- | --- |
| Oat | Filtered Water, Ground Australian Oats, Minerals (Phosphorous, Calcium), Sunflower Oil, Canola Oil, Sea Salt |
| Soy | Filtered Water, Organic Whole Soybeans(Certif, Usda, Nop), Tapioca Syrup, Sea Salt, Job's Tears (Hato Mugi), Calcium Carbonate |
| Almond | Australian water, almonds, sugar, vegetable oil (canola or sunflower), maltodextrin (from corn), acidity regulators (potassium citrate, dipotassium phosphate), vegetable gums, sunflower lecithin, salt. |
| Coconut | Australian water, coconut cream, raw sugar, vegetable protein, salt, vegetable gums, natural flavour |
| Pasteurized cow | Milk |
| UHT Cow | Organic Australian ultra heat-treated milk |
| Formulation 1* | Water (89.11%), olive oil (2.8%), tapioca syrup (2%), soy protein isolate (2%), soy whole bean premix (2%), brown rice (1%), inulin (0.5%), dipotassium phosphate (0.24%), salted caramel (0.1%), xanthan (0.1%), calcium carbonate (0.05%), table salt (0.04%), vanilla flavor (0.03%), yeast extract (0.02%), citric acid (0.01%) |
| Formulation 2* | Water (86.11%), tapioca syrup (5%), olive oil (2.8%), soy protein Isolate (2%), soy whole bean premix (2%), brown rice (1%), inulin (0.5%), dipotassium phosphate (0.24%), salted caramel (0.1%), xanthan (0.1%), calcium carbonate (0.05%), table salt (0.04%), vanilla flavor (0.03%), yeast extract (0.02%), citric acid (0.01%) |

* Formulation 1 was generated using the Random Forest Regression model, while Formulation 2 was generated using the Gradient Boosting Regression model.

**Supplementary Table 2: Attribute list for descriptive analysis, including definitions and references**

| **Attribute** | **Definitions** | **References** |
| --- | --- | --- |
| **Appearance** |  |  |
| White color | The intensity of white color | White colored paper |
| Yellow color | The intensity of yellow color | Yellow colored paper |
| Brown color | The intensity of brown color | Brown colored paper |
|  |  |  |
| **Aroma** |  |  |
| Overall aroma intensity | The overall aroma intensity | n/a |
| Cereal | The intensity of cereal aroma including malty, oat and flour | 55 g of oats in 1000 g of skim milk |
| Beany | The intensity of beany flavor including soybeans, grassy, hay and peas | 180 g of peas in 1000 g of skim milk |
| Dairy | The intensity of dairy aroma including cream, butter and milk | 15 g of cream |
| Nutty | The intensity of the nutty aroma including almonds | 380 g of almond meal plus 2 mL almond extract in 1000 g of skim milk |
| Vanilla | The intensity of vanilla aroma | 50 g of vanilla extract in 1000 g of skim milk |
| Caramel | The intensity of caramel aroma | 90 g salted caramel in 1000 g of skim milk |
| Coconut | The intensity of coconut aroma | 100 g of coconut shreds in 1000 g of skim milk |
| Chocolate | The intensity of the chocolate and cocoa aroma | 70 g of chocolate powder in 1000 g of skim milk |
| **Taste and aftertaste** |  |  |
| Sweetness | The intensity of sweet taste including aftertaste | 9 g of sugar in 1000 g of water |
| Sourness | The intensity of sour taste including aftertaste | 0.225 g of citric acid in 1000 g of water |
| Umami | The intensity of umami taste (savory) including aftertaste | 2 g of Monosodium glutamate in 1000 g of water |
| Bitterness | The intensity of bitter taste including aftertaste | 0.25 g of caffeine in 1000 g of water |
| Saltiness | The intensity of salt taste including aftertaste | 1.35 g of table salt in 1000 g of water |
| Astringency | The intensity of the astringent mouthfeel including drying aftertaste | 8x English breakfast tea bags in 1000 g of water |
| Metallic | The intensity of metallic aftertaste | n/a |
| Coconut | The intensity of coconut aftertaste | 100 g of coconut shreds in 1000 g of skim milk |
| **Flavor** |  |  |
| Overall flavor intensity | The overall aroma intensity | n/a |
| Beany | The intensity of beany flavor including soybeans, grassy, hay and peas | 180 g of peas in 1000 g of skim milk |
| Chocolate | The intensity of the chocolate and cocoa flavor | 70 g of chocolate powder in 1000 g of skim milk |
| Nutty | The intensity of the nutty flavor including almonds |  |
| Metallic | The intensity of metal flavor | n/a |
| Coconut | The intensity of the coconut flavor | 380 g of almond meal plus 2mL almond extract in 1000 g of skim milk |
|  |  |  |
| **Mouthfeel** |  |  |
| Grittiness | The intensity of gritty texture | 6 g of almond meal |
| Creaminess | The intensity of creaminess including fattiness and smoothness of sample | 15 g of cream |
| Viscosity | The intensity of viscous mouthfeel i.e. low = water; high = oil | 1.5 g of carboxymethyl cellulose in 1000 g of water |

**Supplementary Table 3**. Ingredient list for oat, soy, almond, coconut, rice, plant-hybrid, and UHT cow milk types, as per manufacturers' declaration

| **Sample** | **Ingredients** |
| --- | --- |
| Almond 1 | Water, almonds, sugar, canola oil, sunflower oil, maltodextrin, potassium citrate, dipotassium phosphate, gellan gum, xanthan gum, locust bean gum, sunflower lecithin, salt |
| Almond 2 | Water, almonds, sugar, canola oil, sunflower oil, maltodextrin, potassium citrate, dipotassium phosphate, gellan gum, xanthan gum, locust bean gum, calcium carbonate, sunflower lecithin, salt |
| Almond 3 | Water, almonds, calcium phosphate, sunflower lecithin, salt, natural flavour, gellan gum |
| Almond 4 | Water, almonds, sugar, canola oil, sunflower oil, maltodextron, potassium citrate, dipotassium phosphate, gellan gum, xanthan gum, locust bean gum, calcium carbonate, sunflower lecithin, salt |
| Almond 5 | Water, almonds, sugar, calcium phosphate, natural flavour, sunflower lecithin, salt, gellan gum |
| Almond 6 | Water, almonds, calcium, sunflower lecithin, natural flavour, salt, sodium bicarbonate, gellan gum, ascorbic acid, vitamin B12, vitamin B2, vitamin B2 |
| Almond 7 | Water, almonds, calcium carbonate, tapioca starch, salt, carrageenan, soy lecithin, natural flavour |
| Almond 8 | Water, almonds, brown rice, calcium, salt |
| Almond 9 | Water, almonds, calcium carbonate, gellan gum, xanthan gum, cellulose, carboxymethyl cellulose, vanilla flavour, salt, lecithin |
| Almond 10 | Water,almonds, sugar, maltodextrin, sunflower oil, canola oil, potassium phosphate, calcium, lecithin, salt, locust bean gum, gellan gum, ascorbic acid, vitamin B12, vitamin B2, vitamin B1 |
| Coconut 1 | Water, coconut, sugar, vegetable protein, salt, gellan gum, xanthan gum, natural flavour |
| Coconut 2 | Water, coconut, brown rice, salt |
| Coconut 3 | Water, coconut, brown rice, salt |
| Coconut 4 | Coconut, Water, guar gum, 435 emulsifier |
| Coconut 5 | Water, coconut, carboxymethyl cellulose, 435 emulsifier |
| Coconut 6 | Coconut, water, guar gum |
| Coconut 7 | Water, coconut, brown rice, calcium carbonate, salt |
| Coconut 8 | Water, coconut, salt, locust bean gum, sodium alginate, guar gum |
| Coconut 9 | Coconut, water |
| Coconut 10 | Coconut, water |
| Cow 1 | Full cream milk |
| Cow 2 | Full cream milk |
| Cow 3 | Full cream milk |
| Cow 4 | Full cream milk |
| Cow 5 | Full cream milk |
| Cow 6 | Full cream milk |
| Cow 7 | Full cream milk |
| Cow 8 | Full cream milk |
| Cow 9 | Full cream milk |
| Cow 10 | Full cream milk |
| Plant-hybrid | Water, coconut oil, pea protein, chicory fibre, sugar, pineapple juice concentrate, sunflower oil, dipotassium phosphate, calcium carbonate, natural flavour, acacia gum, gellan gum, salt, cabbage juice concentrate |
| Oat 1 | Water, oats, phosphorous, calcium, sunflower oil, canola oil, salt |
| Oat 2 | Water, oats, oleic sunflower, calcium, salt |
| Oat 3 | Water, oats, calcium carbonate, salt |
| Oat 4 | Water, oats, calcium carbonate, salt |
| Oat 5 | Water, oats, canola oil, dipotassium phosphate, calcium carbonate, tricalcium phosphate, salt, gellan gum, vitamin A |
| Oat 6 | Water, oats, rapeseed oil, dipotassium phosphate, calcium, salt |
| Oat 7 | Water, oats, sunflower oil, gum arabic, calcium carbonate,calcium phosphate, potassium phosphate, natural flavour, salt |
| Oat 8 | Water, oats, rapeseed oil, dibasic potassium phosphate, calcium carbonate, salt, vitamin B2, vitamin D2, vitamin B12 |
| Oat 9 | Water, oats, vegetable oils, calcium, phosphorus, salt, natural flavour, ascobic acid, vitamin B12, vitamin B2, vitamin D, vitamin B1 |
| Oat 10 | Water, oats, canola oil, salt |
| Rice 1 | Water, brown rice, sunflower oil, calcium carbonate, salt |
| Rice 2 | Water, brown rice, sunflower oil, calcium phosphate, salt |
| Rice 3 | Water, brown rice, sunflower oil, calcium, salt |
| Soy 1 | Water, soybeans, tapioca syrup, salt, job's tears, calcium carbonate |
| Soy 2 | Water, soy protein, maltodextrin, canola oil, sugar, calcium, phosphate, magnesium carbonate, magnesium bicarbonate, calcium hydroxide, lecithin, calcium chloride, vitamin A, vitamin B12, vitamin B2 |
| Soy 3 | Water, soy, canola oil, maltodextrin, sugar, 332, phosphate, potassium, natural flavour, sunflower lecithin, cellulose, carboxylmethyl cellulose, salt |
| Soy 4 | Water, soy protein, sunflower oil, canola oil, maltodextrin, sugar, calcium, phosphorus, magnesium, vegetable fibre, 332, phosphate, ascorbic acid, vitamin B12, vitamin A, vitamin B2, vitamin B1 |
| Soy 5 | Water, soy, sugar, salt, calcium carbonate |
| Soy 6 | Water, soy protein, maltodextrin, canola oil, sugar, phosphoric acid, calcium hydroxide, potassium citrate, magnesium carbonate, calcium, phosphate, calcium chloride, vitamin A, vitamin B12, vitamin B1 |
| Soy 7 | Water, soy, sunflower oil, calcium, salt |
| Soy 8 | Water, soy, barley malt, barley flavour, sugar, sunflower oil, calcium phosphate, natural flavour, salt, kombu, vitamin A, vitamin B12, vitamin B2 |
| Soy 9 | Water, soy protein, maltodextrin, sugar, sunflower oil, canola oil, vegetable fibre, phosphorus, calcium, magnesium, 332, calcium, phosphate |
| Soy 10 | Water, soy, sugar, sunflower oil, calcium phosphate, natural flavour, salt |

**Supplementary Table 4**. Whiteness index, particle size, viscosity, pH and stability index of oat, soy, almond, coconut, rice, plant-hybrid, and UHT cow milk types

| Sample | Whiteness index | (D_50_) Particle size (um) | Viscosity (mPa·s) | pH | Stability index |
| --- | --- | --- | --- | --- | --- |
| Almond 1 | 68.65102 | 1.922 | 13.4 | 7.42 | 1.919007 |
| Almond 2 | 69.53512 | 1.038 | 2.8 | 7.68 | 0.733569 |
| Almond 3 | 61.14064 | 1.71 | 1.2 | 8.25 | 1.599137 |
| Almond 4 | 63.40106 | 1.58 | 5.1 | 8.05 | 1.801141 |
| Almond 5 | 67.95051 | 1.27 | 1.9 | 8.19 | 0.778626 |
| Almond 6 | 62.93019 | 1.51 | 2.1 | 7.47 | 0.551711 |
| Almond 7 | 64.29854 | 2.99 | 2.5 | 7.71 | 1.405567 |
| Almond 8 | 62.97435 | 1.13 | 0.8 | 7.75 | 2.213863 |
| Almond 9 | 63.6033 | 1.42 | 2.4 | 7.7 | 2.114419 |
| Almond 10 | 72.74417 | 1.85 | 2.1 | 8.03 | 0.401409 |
| Coconut 1 | 74.33686 | 1.93 | 12.8 | 6.38 | 2.096737 |
| Coconut 3 | 65.79066 | 5.95 | 1.9 | 5.88 | 2.339181 |
| Coconut 4 | 75.02869 | 4.546 | 0 | 5.89 | 0 |
| Coconut 5 | 68.26706 | 4.316 | 0 | 6.02 | 0 |
| Coconut 6 | 70.94907 | 2.438 | 0 | 6.39 | 0 |
| Coconut 7 | 67.77488 | 3.792 | 0 | 6.31 | 0 |
| Coconut 8 | 64.90821 | 2.782 | 1.6 | 6.27 | 5.589613 |
| Coconut 9 | 72.21192 | 3.378 | 0 | 5.89 | 0 |
| Coconut 10 | 74.72869 | 5.644 | 0 | 5.93 | 0 |
| Coconut 10 | 76.78714 | 5.186 | 0 | 5.95 | 0 |
| Coconut 10 | 76.72638 | 5.042 | 0 | 5.89 | 0 |
| Coconut 10 | 76.48274 | 4.96 | 0 | 5.93 | 0 |
| Cow 1 | 76.89107 | 1.41 | 1.9 | 6.59 | 2.848269 |
| Cow 2 | 71.88734 | 0.7494 | 2 | 6.92 | 0.494405 |
| Cow 3 | 73.45115 | 0.798 | 1.8 | 6.74 | 6.106617 |
| Cow 4 | 70.28496 | 0.8062 | 1.8 | 6.76 | 0.733108 |
| Cow 5 | 72.60935 | 0.7962 | 2.5 | 6.82 | 0.61781 |
| Cow 6 | 70.72356 | 0.728 | 1.9 | 6.67 | 0.796813 |
| Cow 7 | 72.04926 | 0.80325 | 1.9 | 6.85 | 0.778626 |
| Cow 8 | 71.76201 | 0.797 | 2 | 6.82 | 0.157189 |
| Cow 9 | 74.17267 | 0.7212 | 1.9 | 6.88 | 0.808118 |
| Cow 10 | 76.87515 | 0.7156 | 2.5 | 6.78 | 1.199327 |
| Plant-hybrid | 72.34971 | 1.04 | 1.6 | 7.43 | 2.393104 |
| Oat 1 | 69.63191 | 1.03 | 1.4 | 6.9 | 2.851078 |
| Oat 2 | 64.8279 | 1.254 | 2.1 | 7.35 | 4.250879 |
| Oat 3 | 63.1744 | 1.4896 | 2.1 | 6.98 | 5.727952 |
| Oat 4 | 66.25033 | 0.8576 | 2 | 7.04 | 2.273992 |
| Oat 5 | 73.00564 | 1.644 | 5.1 | 7.19 | 0.264394 |
| Oat 6 | 70.7469 | 0.8252 | 1.5 | 7.22 | 0.512793 |
| Oat 7 | 62.72306 | 1.436 | 1.6 | 7.49 | 1.293843 |
| Oat 8 | 58.62382 | 1.566 | 2.7 | 7.7 | 5.33997 |
| Oat 9 | 69.0569 | 0.999 | 1.2 | 7.32 | 1.970806 |
| Oat 10 | 69.62432 | 3.474 | 1.4 | 6.62 | 8.953057 |
| Rice 1 | 69.63191 | 4.22 | 2.8 | 6.82 | 18.74705 |
| Rice 2 | 64.47468 | 1.092 | 1.6 | 6.83 | 2.153069 |
| Rice 3 | 60.27816 | 3.05 | 1.4 | 6.68 | 9.951608 |
| Soy 1 | 71.73604 | 1.89 | 3.5 | 6.68 | 2.527072 |
| Soy 2 | 71.26995 | 0.8914 | 2.5 | 7.11 | 0.806866 |
| Soy 3 | 68.52412 | 0.8726 | 2.6 | 7.09 | 1.185571 |
| Soy 4 | 68.5354 | 0.8064 | 1.8 | 6.68 | 4.270269 |
| Soy 5 | 64.30904 | 1.152 | 1.9 | 7.98 | 2.284013 |
| Soy 6 | 72.06966 | 0.9818 | 2.4 | 7.38 | 0.457831 |
| Soy 7 | 67.38309 | 0.8624 | 1.5 | 7.4 | 0.644444 |
| Soy 8 | 71.57299 | 1.274 | 3.8 | 7.06 | 0.612183 |
| Soy 9 | 73.00368 | 0.7798 | 1.4 | 7.64 | 0.586503 |
| Soy 10 | 70.38051 | 0.9908 | 2.6 | 7.39 | 0.362863 |

**Supplementary Table 5**. Chemical composition of oat, soy, almond, coconut, rice, plant-hybrid, and UHT cow milk types

| Sample | Protein (g) | Carbohydrates (g) | Sugar(g) | Sodium (mg) | Dietary fibre (g) | Total fat (g) | Calcium (mg) |
| --- | --- | --- | --- | --- | --- | --- | --- |
| Almond 1 | 0.8 | 2.8 | 1.7 | 41 | 0 | 2.5 | 0 |
| Almond 2 | 0.6 | 4.8 | 2.1 | 64 | 0 | 2.4 | 0 |
| Almond 3 | 0.7 | 0.3 | 0.1 | 36 | 0.3 | 1.8 | 0 |
| Almond 4 | 0.8 | 2.8 | 1.7 | 40 | 0 | 2.5 | 0 |
| Almond 5 | 0.6 | 2.0 | 1.9 | 59 | 0.3 | 2.7 | 0 |
| Almond 6 | 0.6 | 0.3 | 0.1 | 36 | 0.3 | 1.4 | 0 |
| Almond 7 | 0.5 | 0.7 | 0.1 | 52 | 0.3 | 1.2 | 0 |
| Almond 8 | 0.7 | 2.6 | 1.1 | 37 | 0.3 | 1.78 | 0 |
| Almond 9 | 1.0 | 1.0 | 1.0 | 41 | 1.0 | 1.2 | 120 |
| Almond 10 | 0.8 | 2.8 | 1.6 | 39 | 0.5 | 2.6 | 0 |
| Coconut 1 | 0.6 | 3.0 | 2.6 | 48 | 0 | 3.7 | 0 |
| Coconut 2 | 0.2 | 0.5 | 0.2 | 44 | 0.2 | 1.8 | 0 |
| Coconut 3 | 0.5 | 7.8 | 3.8 | 61 | 0 | 2.7 | 0 |
| Coconut 4 | 0.3 | 2.6 | 1.9 | 40 | 0 | 18.3 | 0 |
| Coconut 5 | 0.6 | 2.6 | 1.0 | 39 | 0 | 6.0 | 0 |
| Coconut 6 | 1.7 | 3.2 | 2.1 | 31 | 0 | 2.1 | 0 |
| Coconut 7 | 1.0 | 6.6 | 3.1 | 45 | 1.0 | 1.8 | 120 |
| Coconut 8 | 0.1 | 0.2 | 0.2 | 46 | 0 | 1.8 | 0 |
| Coconut 9 | 2.6 | 3.9 | 1.6 | 24 | 0.3 | 24.3 | 0 |
| Coconut 10 | 1.6 | 2.4 | 1.7 | 15 | 0.3 | 17.1 | 0 |
| Cow 1 | 3.2 | 4.8 | 4.8 | 45 | 0 | 3.4 | 120 |
| Cow 2 | 3.7 | 5.1 | 5.1 | 40 | 0 | 3.6 | 135 |
| Cow 3 | 3.4 | 4.8 | 4.8 | 49 | 0 | 3.6 | 118 |
| Cow 4 | 3.6 | 4.5 | 4.2 | 40 | 0 | 3.9 | 125 |
| Cow 5 | 3.3 | 4.8 | 4.8 | 40 | 0 | 3.4 | 119 |
| Cow 6 | 3.3 | 4.8 | 4.8 | 41 | 0 | 3.6 | 120 |
| Cow 7 | 3.3 | 4.8 | 4.8 | 40 | 0 | 3.4 | 120 |
| Cow 8 | 3.3 | 4.8 | 4.8 | 40 | 0 | 3.4 | 125 |
| Cow 9 | 3.4 | 5.3 | 4.8 | 40 | 0 | 3.4 | 120 |
| Cow 10 | 3.3 | 4.8 | 4.8 | 40 | 0 | 3.4 | 117 |
| Plant-hybrid | 1.6 | 1.8 | 1.2 | 62 | 0 | 3.3 | 0 |
| Oat 1 | 0.8 | 6.1 | 1.8 | 48 | 0.4 | 3.0 | 120 |
| Oat 2 | 1.4 | 7.7 | 3.5 | 45 | 1.0 | 1.3 | 120 |
| Oat 3 | 1.5 | 8.3 | 3.9 | 45 | 1.0 | 1.0 | 120 |
| Oat 4 | 0.6 | 7.0 | 2.8 | 55 | 0.5 | 2.8 | 120 |
| Oat 5 | 0.9 | 6.3 | 4.4 | 43 | 0.9 | 4.0 | 120 |
| Oat 6 | 0.8 | 5.8 | 2.8 | 50 | 0.8 | 2.9 | 50 |
| Oat 7 | 0.6 | 6.3 | 1.1 | 50 | 0.9 | 2.1 | 120 |
| Oat 8 | 1.1 | 7.1 | 3.4 | 40 | 0.8 | 3.0 | 120 |
| Oat 9 | 0.8 | 6.3 | 2.0 | 45 | 0.4 | 1.9 | 120 |
| Oat 10 | 1.1 | 7.7 | 2.0 | 36 | 0 | 1.9 | 0 |
| Rice 1 | 0.6 | 10.3 | 3.6 | 68 | 1.0 | 1.2 | 100 |
| Rice 2 | 0.3 | 9.5 | 5.8 | 65 | 0.2 | 1.2 | 120 |
| Rice 3 | 0.6 | 9.0 | 3.1 | 65 | 4.0 | 1.0 | 120 |
| Soy 1 | 4.1 | 5.5 | 2.2 | 47 | 1.4 | 2.2 | 25 |
| Soy 2 | 3.6 | 5.0 | 1.6 | 60 | 0.2 | 3.3 | 120 |
| Soy 3 | 3.3 | 5.0 | 1.9 | 85 | 0 | 3.0 | 0 |
| Soy 4 | 3.2 | 4.2 | 1.9 | 47 | 0.7 | 3.0 | 120 |
| Soy 5 | 3.9 | 2.6 | 1.5 | 93 | 0 | 2.5 | 24 |
| Soy 6 | 3.2 | 5.4 | 5.4 | 60 | 0 | 3.2 | 110 |
| Soy 7 | 3.0 | 1.5 | 0.8 | 15 | 1.0 | 3.4 | 120 |
| Soy 8 | 3.3 | 8.0 | 2.6 | 60 | 0.6 | 3.0 | 0 |
| Soy 9 | 3.2 | 4.3 | 1.9 | 47 | 0 | 3.0 | 57 |
| Soy 10 | 6.0 | 3.4 | 2.5 | 80 | 0.2 | 3.0 | 0 |

**Supplementary Table 6**. Volatile compounds and their integrated peak areas identified in oat, soy, almond, coconut, rice, plant-hybrid, and UHT cow milk types

| Sample | Isobutanol | 2,3-Methylbutanol | 1-Butanol | 1-Butanol, 2,3-dimethyl- | 1-Butanol, 3-methyl | 1-Butanol, 2-methyl- | 1-Dodecanol | 1-Heptanol | 1-Hexanol |
| --- | --- | --- | --- | --- | --- | --- | --- | --- | --- |
| Almond 1 | 0 | 0 | 0 | 0 | 0 | 0 | 0 | 0 | 0 |
| Almond 2 | 0 | 0 | 0 | 0 | 0 | 0 | 0 | 0 | 0 |
| Almond 3 | 0 | 0 | 0 | 0 | 0 | 0 | 0 | 0 | 0 |
| Almond 4 | 0 | 0 | 0 | 366827 | 0 | 222085 | 0 | 136113 | 1348832 |
| Almond 5 | 0 | 0 | 0 | 0 | 0 | 0 | 0 | 0 | 0 |
| Almond 6 | 0 | 0 | 0 | 0 | 0 | 0 | 0 | 132219 | 1102808 |
| Almond 7 | 0 | 0 | 0 | 0 | 0 | 0 | 0 | 0 | 427301 |
| Almond 8 | 0 | 0 | 0 | 0 | 67842 | 0 | 0 | 0 | 1158409 |
| Almond 9 | 0 | 0 | 0 | 0 | 0 | 62650 | 0 | 416152 | 2055932 |
| Almond 10 | 0 | 0 | 0 | 0 | 0 | 0 | 0 | 0 | 712576.5 |
| Coconut 1 | 0 | 0 | 0 | 45714 | 0 | 221630 | 99572 | 0 | 0 |
| Coconut 2 | 0 | 0 | 0 | 0 | 0 | 0 | 0 | 0 | 0 |
| Coconut 3 | 0 | 0 | 74179 | 151026 | 0 | 546892 | 0 | 0 | 1498669 |
| Coconut 4 | 0 | 0 | 950589.5 | 0 | 309731 | 183668 | 0 | 0 | 0 |
| Coconut 5 | 0 | 0 | 567953 | 0 | 0 | 0 | 0 | 0 | 139459 |
| Coconut 6 | 0 | 0 | 483312.5 | 0 | 967450 | 0 | 0 | 0 | 311340.5 |
| Coconut 7 | 0 | 0 | 637810.5 | 0 | 0 | 1050712 | 0 | 0 | 208333 |
| Coconut 8 | 0 | 0 | 0 | 0 | 344065.5 | 0 | 0 | 0 | 0 |
| Coconut 9 | 0 | 0 | 491624 | 0 | 365308 | 0 | 0 | 0 | 271853 |
| Coconut 10 | 0 | 0 | 1055692 | 0 | 0 | 0 | 0 | 0 | 307042.5 |
| Cow 1 | 0 | 0 | 0 | 0 | 283185 | 0 | 0 | 354183 | 0 |
| Cow 2 | 0 | 0 | 0 | 0 | 0 | 0 | 0 | 0 | 0 |
| Cow 3 | 0 | 0 | 0 | 0 | 0 | 0 | 0 | 0 | 0 |
| Cow 4 | 0 | 0 | 0 | 0 | 0 | 0 | 0 | 0 | 0 |
| Cow 5 | 0 | 0 | 0 | 162374 | 526272 | 0 | 0 | 0 | 0 |
| Cow 6 | 0 | 0 | 0 | 0 | 129277 | 0 | 0 | 0 | 0 |
| Cow 7 | 0 | 0 | 0 | 0 | 0 | 0 | 0 | 0 | 0 |
| Cow 8 | 0 | 0 | 0 | 0 | 341331 | 0 | 0 | 0 | 0 |
| Cow 9 | 0 | 0 | 0 | 0 | 0 | 0 | 0 | 0 | 0 |
| Cow 10 | 0 | 0 | 0 | 0 | 182317 | 0 | 0 | 0 | 0 |
| Plant-hybrid | 34990 | 17027 | 33656 | 0 | 0 | 0 | 0 | 72112 | 0 |
| Oat 1 | 0 | 0 | 0 | 0 | 0 | 0 | 0 | 0 |  |
| Oat 2 | 0 | 0 | 0 | 0 | 0 | 0 | 0 | 606000.5 | 2398714 |
| Oat 3 | 0 | 0 | 0 | 0 | 0 | 0 | 0 | 1185780 | 625218 |
| Oat 4 | 0 | 0 | 0 | 0 | 305445 | 0 | 0 | 0 | 127205 |
| Oat 5 | 0 | 0 | 0 | 0 | 0 | 0 | 0 | 0 | 604389.5 |
| Oat 6 | 0 | 0 | 0 | 0 | 0 | 0 | 0 | 0 | 742458.5 |
| Oat 7 | 0 | 0 | 0 | 0 | 0 | 0 | 0 | 0 | 0 |
| Oat 8 | 0 | 0 | 0 | 0 | 0 | 0 | 0 | 0 | 0 |
| Oat 9 | 0 | 0 | 0 | 0 | 0 | 0 | 0 | 0 | 0 |
| Oat 10 | 0 | 0 | 0 | 0 | 0 | 0 | 0 | 0 | 0 |
| Rice 1 | 0 | 0 | 0 | 0 | 166343 | 0 | 0 | 326101 | 0 |
| Rice 2 | 0 | 0 | 0 | 0 | 213957 | 0 | 0 | 0 | 369748 |
| Rice 3 | 0 | 0 | 0 | 0 | 38000 | 0 | 0 | 0 | 1293961 |
| Soy 1 | 0 | 0 | 0 | 0 | 0 | 0 | 0 | 0 | 0 |
| Soy 2 | 0 | 0 | 0 | 0 | 0 | 0 | 0 | 0 | 1812988 |
| Soy 3 | 0 | 0 | 0 | 0 | 0 | 0 | 0 | 0 | 634814.5 |
| Soy 4 | 0 | 0 | 0 | 0 | 0 | 0 | 0 | 0 | 0 |
| Soy 5 | 0 | 0 | 0 | 0 | 0 | 0 | 0 | 0 | 385124.5 |
| Soy 6 | 0 | 0 | 0 | 0 | 0 | 0 | 0 | 0 | 451027 |
| Soy 7 | 0 | 0 | 0 | 233855 | 0 | 0 | 0 | 0 | 534384.5 |
| Soy 8 | 0 | 0 | 0 | 0 | 0 | 78374 | 0 | 0 | 357967.5 |
| Soy 9 | 0 | 0 | 0 | 0 | 0 | 0 | 0 | 0 | 0 |
| Soy 10 | 0 | 0 | 38976 | 0 | 18278 | 0 | 0 | 0 | 247964 |

**Supplementary Table 6.** continued

| Sample | 1-Octanol | 1-Octen-3-ol | 2-Furanmethanol | 1-Pentanol | 2-Heptanal | 2-Heptanol | 2-Heptanone | 2-Nonanone | Heptanal |
| --- | --- | --- | --- | --- | --- | --- | --- | --- | --- |
| Almond 1 | 0 | 0 | 0 | 0 | 0 | 0 | 3942909 | 651425 | 0 |
| Almond 2 | 0 | 0 | 1076487 | 69378 | 0 | 0 | 0 | 0 | 152370.5 |
| Almond 3 | 0 | 0 | 0 | 0 | 0 | 0 | 0 | 0 | 0 |
| Almond 4 | 0 | 164533.5 | 747628.5 | 410240 | 0 | 0 | 627754.5 | 0 | 571640 |
| Almond 5 | 0 | 0 | 0 |  | 0 | 0 | 0 | 0 | 331600 |
| Almond 6 | 0 | 148294.5 | 0 | 287186 | 0 | 0 | 361341 | 0 | 367017.3 |
| Almond 7 | 0 | 210430.5 | 0 | 461457.5 | 0 | 0 | 152265 | 0 | 99051 |
| Almond 8 | 0 | 318651 | 0 | 636433 | 37976 | 0 | 516636 | 0 | 268040.5 |
| Almond 9 | 0 | 318969 | 0 | 568489 | 74944 | 0 | 594225 | 0 | 79645.5 |
| Almond 10 | 0 | 0 | 125671 | 105747.5 | 0 | 71165 | 0 | 0 | 0 |
| Coconut 1 | 87367 | 0 | 0 | 130329 | 0 | 0 | 0 | 0 | 76986.5 |
| Coconut 2 | 0 | 0 | 0 |  | 0 | 0 | 0 | 127590 | 146539 |
| Coconut 3 | 0 | 237357.5 | 0 | 1294623 | 106316 | 0 | 1708979 | 0 | 622712 |
| Coconut 4 | 462573 | 77528 | 0 |  | 0 | 1393041 | 1490919 | 521307.5 | 0 |
| Coconut 5 | 176516 | 133459 | 0 | 49488 | 0 | 234399 | 816087 | 842362 | 0 |
| Coconut 6 | 289757 | 70831 | 0 | 810725 | 0 | 362038 | 1261095 | 1570752 | 0 |
| Coconut 7 | 89357 | 0 | 0 | 0 | 0 | 95065 | 401469.5 | 875518.5 | 128994 |
| Coconut 8 | 0 | 0 | 0 | 0 | 0 | 0 | 0 | 0 | 0 |
| Coconut 9 | 172010.5 | 0 | 0 | 117330 | 0 | 210371.5 | 362811.5 | 222938 | 0 |
| Coconut 10 | 0 | 0 | 244197 | 1506342 | 0 | 457553 | 841615 | 790343.5 | 0 |
| Cow 1 | 221870 | 0 | 650680 | 1133346 | 0 | 0 | 0 | 0 | 0 |
| Cow 2 | 0 | 0 | 0 | 0 | 0 | 0 | 10538165 | 1394735 | 0 |
| Cow 3 | 0 | 0 | 0 | 0 | 0 | 0 | 10538165 | 1394735 | 0 |
| Cow 4 | 0 | 0 | 0 | 89322 | 0 | 0 | 1585101 | 299547.5 | 0 |
| Cow 5 | 0 | 0 | 65466 | 45634 | 0 | 0 | 2539279 | 310873.7 | 0 |
| Cow 6 | 0 | 0 | 0 | 0 | 0 | 0 | 14447550 | 2255488 | 0 |
| Cow 7 | 0 | 0 | 0 | 0 | 0 | 0 | 9630069 | 1699914 | 0 |
| Cow 8 | 0 | 0 | 0 | 0 | 0 | 0 | 0 | 0 | 37141 |
| Cow 9 | 0 | 0 | 0 | 0 | 0 | 0 | 12578082 | 2234746 | 0 |
| Cow 10 | 0 | 0 | 0 | 0 | 0 | 0 | 9231286 | 1330184 | 0 |
| Plant-hybrid | 60814 | 163804 | 0 | 483500 | 0 | 0 | 8151456 | 1576879 | 0 |
| Oat 1 | 0 | 52991 | 0 | 92580 | 0 | 0 | 5301473 | 262201 | 385411.7 |
| Oat 2 | 0 | 663489.5 | 0 | 1670915 | 121711.5 | 0 |  | 0 | 852703.3 |
| Oat 3 | 0 | 1445427 | 0 | 2353352 | 176835.5 | 0 | 508265.5 | 0 | 1989654 |
| Oat 4 | 0 | 0 | 15543336 | 277251.5 | 0 | 0 | 1033207 | 0 | 256942.3 |
| Oat 5 | 0 | 134021 | 1067865 | 643260 | 0 | 0 | 69604 | 0 | 174119 |
| Oat 6 | 0 | 0 | 10246008 | 264224 | 0 | 0 | 297847.5 | 0 | 0 |
| Oat 7 | 0 | 0 | 2978148 | 89994 | 0 | 0 | 272201 | 0 | 79056 |
| Oat 8 | 0 | 0 | 2495710 | 118877.5 | 0 | 0 | 153872.5 | 0 | 0 |
| Oat 9 | 0 | 0 | 1404624 | 0 | 0 | 0 | 128293 | 0 | 149405 |
| Oat 10 | 0 | 240851 | 0 | 1124472 | 690563 | 0 | 0 | 0 | 0 |
| Rice 1 | 258697 | 534904 | 0 | 1166088 | 0 | 0 | 0 | 0 | 0 |
| Rice 2 | 0 | 130548 | 330253 | 139357 | 67932 | 0 | 0 | 79037 | 1287394 |
| Rice 3 | 259126 | 449159 | 0 | 1741117 | 0 | 0 | 0 | 0 | 0 |
| Soy 1 | 0 | 126372 | 0 | 245960 | 64127 | 0 | 1378730 | 0 | 0 |
| Soy 2 | 0 | 335224 | 172111.5 | 842184.5 | 94102.5 | 0 | 0 | 0 | 0 |
| Soy 3 | 0 | 519647.5 | 523272.5 | 935687.5 | 0 | 0 | 2854013 | 0 | 0 |
| Soy 4 | 0 | 97485 | 0 | 99518 | 0 | 0 | 6396696 | 0 | 0 |
| Soy 5 | 0 | 114344 | 0 | 70020 | 0 | 0 | 2039314 | 0 | 0 |
| Soy 6 | 0 | 262591 | 155805 | 638220 | 0 | 0 | 0 | 281481 | 0 |
| Soy 7 | 0 | 0 | 0 | 95534 | 0 | 0 | 5533232 | 1576879 | 0 |
| Soy 7 | 0 | 0 | 0 | 95534 | 0 | 0 | 0 | 0 | 0 |
| Soy 8 | 0 | 141442.5 | 0 | 164133.5 | 0 | 0 | 120645.5 | 0 | 0 |
| Soy 9 | 0 | 96891.5 | 0 | 94201 | 0 | 0 | 1237871 | 0 | 0 |
| Soy 10 | 0 | 46752 | 0 | 138673.5 | 0 | 0 | 54596 | 0 | 0 |

**Supplementary Table 6**. continued

| Sample | 2-Octanol | 2-Butanone | 3-Octen-2-one | Benzaldehyde | Butanal, 3-methyl- | Butanal, 2-methyl- | Furan, 2-pentyl- | Furfural | Hexanal | Nonanal |
| --- | --- | --- | --- | --- | --- | --- | --- | --- | --- | --- |
| Almond 1 | 130060 | 0 | 0 |  | 0 | 0 | 0 | 0 | 0 | 217485 |
| Almond 2 | 0 | 0 | 0 | 210404.5 | 0 | 0 | 76189 | 108403.5 | 1448112 | 154044.8 |
| Almond 3 | 0 | 0 | 0 |  | 0 | 0 | 0 | 0 | 0 | 0 |
| Almond 4 | 250936 | 0 | 0 | 8503317 | 0 | 5124247 | 184412 | 198153 | 6316410 | 445400 |
| Almond 5 | 0 | 0 | 0 |  | 0 | 0 | 119809 | 174613.5 | 5161513 | 376860 |
| Almond 6 | 148351.8 | 0 | 0 | 11688549 | 0 | 4900665 | 419367 | 67996.5 | 11396755 | 466856.5 |
| Almond 7 | 339118 | 0 | 0 | 1103964 | 0 | 3638475 | 363771.3 |  | 3646206 | 211521.5 |
| Almond 8 | 0 | 0 | 0 | 542175 | 523658 | 0 | 222306.3 | 216971.5 | 7175535 | 619539 |
| Almond 9 | 251084.5 | 0 | 0 | 14939450 | 0 | 6238910 | 88391.5 | 0 | 1615347 | 117216 |
| Almond 10 | 0 | 0 | 0 | 973323 | 0 | 665952 | 62114 | 0 | 2593219 | 168835 |
| Coconut 1 | 87534 | 0 | 0 | 311270 | 191889 | 0 | 0 | 0 | 4396224 | 171481.5 |
| Coconut 2 | 132867 | 0 | 0 | 170913.5 | 0 | 0 | 478422 | 117462.5 | 11546858 | 390665 |
| Coconut 3 | 0 | 0 | 0 | 885276.5 | 1549552 | 0 | 0 | 95203.5 | 1709798 | 368232.3 |
| Coconut 4 | 0 | 0 | 0 | 375221 | 0 | 0 | 81245.33 | 198697.5 | 2109745 | 430621 |
| Coconut 5 | 122639 | 0 | 0 | 459277.5 | 0 | 975381 | 70964 | 209823.5 | 820355.5 | 0 |
| Coconut 6 | 91197 | 0 | 0 | 645181 | 0 | 1798927 | 0 | 137897.5 | 853675.5 | 363016 |
| Coconut 7 | 0 | 0 | 0 | 173559.5 | 0 | 0 | 36466.5 | 0 | 4473567 | 164407.5 |
| Coconut 8 | 0 | 0 | 0 | 140695 | 0 | 0 | 41843 | 0 | 897181 | 263565.5 |
| Coconut 9 | 171073 | 0 | 0 | 305785 | 769989 | 0 | 0 | 0 | 492413 | 237087.5 |
| Coconut 10 | 0 | 0 | 0 | 89987.5 | 0 | 827470 | 0 | 0 | 2401453 | 331882 |
| Cow 1 | 352902 | 0 | 0 | 10492643 | 0 | 4375315 | 0 | 0 | 0 | 0 |
| Cow 2 | 0 | 839098.5 | 0 | 93178 | 0 | 0 | 0 | 0 | 284047 | 0 |
| Cow 2 | 0 | 839098.5 | 0 | 93178 | 0 | 0 | 0 | 0 | 0 | 107514 |
| Cow 3 | 108768 | 413695.5 | 0 | 136407 | 0 | 0 | 0 | 0 | 0 | 0 |
| Cow 4 | 119039 | 2194400 | 0 |  | 0 | 0 | 0 | 0 | 0 | 628540 |
| Cow 5 | 147681 | 1446823 | 0 | 90132.5 | 0 | 0 | 0 | 0 | 44016 | 372047 |
| Cow 6 | 82653 | 924242 | 0 | 81473 | 0 | 0 | 0 | 0 | 0 | 0 |
| Cow 7 | 0 | 0 | 0 | 30840 | 0 | 0 | 0 | 0 | 40614 | 323448 |
| Cow 8 | 191652 | 1200946 | 0 | 95305 | 0 | 0 | 0 | 0 | 0 | 0 |
| Cow 9 | 0 | 1116798 | 0 | 70492 | 0 | 0 | 2655094 | 0 | 8014487 | 0 |
| Cow 10 | 85891 | 1065808 | 0 |  | 0 | 0 | 201346 | 0 | 505613 | 143795 |
| Plant-hybrid | 0 | 0 | 92283 | 424451 | 0 | 0 | 1418853 | 0 | 6608829 | 551174.5 |
| Oat 1 | 0 | 0 | 0 | 102480 | 1005744 | 0 | 2922617 | 0 | 13139765 | 1713301 |
| Oat 2 | 283397 | 0 | 53947 | 814701.5 | 1565317 | 0 | 412646.7 | 747709 | 3543650 | 209410 |
| Oat 3 | 350399 | 0 | 120558.5 | 1621360 | 754500 | 0 | 217263.8 | 74565 | 7323641 | 262625.5 |
| Oat 4 | 153287 | 0 | 77904 | 248149 | 4157176 | 0 | 2878479 | 416139.5 | 1675606 | 88203.5 |
| Oat 5 | 181360 | 0 | 54575 | 494953.5 | 4733850 | 0 | 139839.5 | 52971 | 0 | 80116 |
| Oat 6 | 188484.5 | 0 | 0 | 236569 |  | 2371876 | 261228 | 105693.5 | 2065197 | 137290.5 |
| Oat 7 | 169685.5 | 0 | 0 | 100334 | 2478288 | 3471178 | 164236.2 | 60451 | 1436130 | 141535 |
| Oat 8 | 197660 | 0 | 0 | 128878.5 | 964533.5 | 0 | 676115 | 0 | 31535789 | 574061 |
| Oat 9 | 322564 | 0 | 0 | 121825.5 | 1471420 | 0 | 766221 | 0 | 113073 | 476624 |
| Oat 10 | 0 | 20584 | 1431485 | 217648.5 | 1695351 | 0 | 105971.5 | 0 | 2737986 | 308757 |
| Rice 1 | 0 | 0 | 87444 | 1005826 | 356660 | 0 | 519313 | 0 | 8354585 | 452749 |
| Rice 2 | 155580 | 0 | 0 | 100875 | 0 | 0 | 177125 | 54725 | 54725 | 95756 |
| Rice 3 | 266804 | 0 | 84535 | 1285117 | 1203567 | 0 | 2147069 | 0 | 14838696 | 105169.5 |
| Soy 1 | 0 | 0 | 0 | 246551 | 0 | 0 | 3635944 | 0 | 14779613 | 198204.5 |
| Soy 2 | 0 | 505386 | 0 | 325407 | 0 | 0 | 1078766 | 0 | 3757030 | 47232.5 |
| Soy 3 | 0 | 704159.5 | 76217.5 | 1703098 | 0 | 0 | 146888.5 | 0 | 620233 | 27812 |
| Soy 4 | 86828 | 0 | 0 | 193136.5 | 0 | 0 | 2094371 | 0 | 0 | 165274 |
| Soy 5 | 70158 | 0 | 0 | 248101.5 | 0 | 0 | 56896.5 | 139642 | 1390201 | 70287.67 |
| Soy 6 | 85891 | 897243.5 | 112045 | 796118 | 0 | 0 | 88785 | 79822 | 0 | 125753.5 |
| Soy 7 | 229515 | 0 | 0 | 254240.5 | 0 | 1385868 | 779709.2 | 0 | 0 | 50141.5 |
| Soy 8 | 123947 | 0 | 0 | 299490 | 675716.5 | 0 | 141473.5 | 0 | 0 | 103536 |
| Soy 9 | 97258 | 352590 | 0 | 189370 | 178096 | 0 | 0 | 0 | 0 | 0 |
| Soy 10 | 137209 | 0 | 0 | 191471.5 | 0 | 0 | 0 | 0 | 0 | 0 |

**Supplementary Table 6.** continued

| Sample | Octanal | Pentanal | Pyrazine, 2,5-dimethyl- | Pyrazine, 2,6-dimethyl- | Vanillin | δ-Octalactone | δ-Decalactone | δ-undecalactone |
| --- | --- | --- | --- | --- | --- | --- | --- | --- |
| Almond 1 | 0 | 0 | 0 | 0 | 0 | 0 | 0 | 0 |
| Almond 2 | 138191 | 0 | 49479.5 | 45099 | 0 | 0 | 0 | 0 |
| Almond 3 | 0 | 0 | 0 | 0 | 0 | 0 | 0 | 0 |
| Almond 4 | 350613.7 | 311922 | 382873.5 | 76632 | 0 | 0 | 0 | 0 |
| Almond 6 | 172165 | 518704 | 353624 | 75150 | 0 | 0 | 0 | 0 |
| Almond 7 | 230637 | 972263.5 | 238676 | 0 | 0 | 0 | 0 | 0 |
| Almond 8 | 282681 | 0 | 0 | 0 | 0 | 0 | 0 | 0 |
| Almond 9 | 404669 | 839765 | 425950 | 116158 | 0 | 0 | 0 | 0 |
| Almond 10 | 200163 | 0 | 0 | 0 | 0 | 0 | 588290 | 0 |
| Coconut 1 | 45264 | 0 | 0 | 0 | 0 | 0 | 0 | 0 |
| Coconut 2 | 0 | 0 | 0 | 0 | 0 | 657215 | 353639 | 399057 |
| Coconut 3 | 403402 | 1356350 | 0 | 0 | 0 | 210088 | 384847.5 | 0 |
| Coconut 4 | 268405 | 0 | 0 | 0 | 0 | 2524601 | 494405.5 | 0 |
| Coconut 5 | 224823 | 0 | 0 | 0 | 0 | 2278027 | 507589.5 | 0 |
| Coconut 6 | 0 | 0 | 0 | 154782.5 | 0 | 756198.5 | 674904.5 | 0 |
| Coconut 7 | 100367 | 0 | 90509 | 99362 | 0 | 748830 | 38940 | 0 |
| Coconut 8 | 48317 | 0 | 0 | 0 | 0 | 1166497 | 673882 | 0 |
| Coconut 9 | 44998 | 0 | 0 | 0 | 0 | 496742 | 568482.5 | 0 |
| Coconut 10 | 0 | 0 | 0 | 39970 | 0 | 772209.5 | 489575.5 | 0 |
| Cow 1 | Octanal | 0 | 1508273 | 387556 | 0 | 2174490 | 885255 | 0 |
| Cow 2 | 0 | 0 | 0 | 0 | 0 | 0 | 0 | 0 |
| Cow 3 | 0 | 0 | 0 | 0 | 0 | 0 | 54673 | 0 |
| Cow 4 | 19048 | 0 | 0 | 0 | 0 | 86718.5 | 0 | 0 |
| Cow 5 | 0 | 0 | 0 | 0 | 49259 | 0 | 76754 | 0 |
| Cow 6 | 82576 | 0 | 0 | 0 | 0 | 0 | 102865.5 | 0 |
| Cow 7 | 24832 | 0 | 0 | 0 | 0 | 0 | 0 | 0 |
| Cow 8 | 0 | 0 | 0 | 0 | 0 | 0 | 0 | 0 |
| Cow 9 | 0 | 0 | 0 | 0 | 0 | 0 | 104038 | 0 |
| Cow 10 | 0 | 0 | 0 | 0 | 59794 | 0 | 93513 | 0 |
| Plant-hybrid | 117728 | 1374125 | 1196814 | 724736 | 0 | 0 | 0 | 0 |
| Oat 1 | 23230 | 0 | 0 | 0 | 0 | 0 | 0 | 0 |
| Oat 2 | 506652 | 0 | 0 | 0 | 0 | 0 | 0 | 0 |
| Oat 3 | 1410999 | 877473.3 | 0 | 0 | 101715 | 0 | 0 | 0 |
| Oat 4 | 173129 | 0 | 0 | 0 | 0 | 0 | 0 | 0 |
| Oat 5 | 272450 | 0 | 0 | 0 | 0 | 0 | 0 | 0 |
| Oat 6 | 429344 | 0 | 159207 | 703917.5 | 0 | 0 | 0 | 0 |
| Oat 7 | 0 | 0 | 0 | 0 | 0 | 0 | 0 | 0 |
| Oat 8 | 0 | 0 | 0 | 0 | 63059 | 0 | 0 | 0 |
| Oat 9 | 0 | 0 | 0 | 0 | 0 | 0 | 0 | 0 |
| Oat 10 | 410986 | 2558930 | 0 | 0 | 118327 | 0 | 0 | 0 |
| Rice 1 | 183562 | 1281130 | 0 | 0 | 0 | 0 | 0 | 0 |
| Rice 2 | 130679 | 0 | 0 | 0 | 0 | 0 | 0 | 0 |
| Rice 3 | 403209 | 0 | 0 | 0 | 0 | 0 | 0 | 0 |
| Soy 1 | 0 | 0 | 0 | 0 | 0 | 0 | 0 | 0 |
| Soy 2 | 0 | 0 | 0 | 0 | 0 | 0 | 0 | 0 |
| Soy 3 | 0 | 0 | 0 | 0 | 74337 | 0 | 0 | 0 |
| Soy 4 | 0 | 0 | 107414.5 | 0 | 35311 | 0 | 0 | 0 |
| Soy 5 | 0 | 0 | 33153 | 0 | 0 | 0 | 0 | 0 |
| Soy 6 | 267415 | 0 | 0 | 0 | 0 | 0 | 0 | 0 |
| Soy 7 | 74277 | 0 | 130798 | 0 | 0 | 0 | 0 | 0 |
| Soy 8 | 0 | 0 | 0 | 0 | 0 | 0 | 0 | 0 |
| Soy 9 | 0 | 0 | 83867.5 | 0 | 0 | 0 | 0 | 0 |
| Soy 10 | 0 | 0 | 0 | 0 | 0 | 0 | 0 | 0 |

**Supplementary Table 7**. The pH values of oat, soy, almond, coconut, rice, plant-hybrid, and UHT cow milk types

| Sample | pH |
| --- | --- |
| Almond 1 | 7.43 |
| Almond 2 | 7.68 |
| Almond 3 | 8.25 |
| Almond 4 | 8.05 |
| Almond 5 | 8.19 |
| Almond 6 | 7.46 |
| Almond 7 | 7.73 |
| Almond 8 | 7.73 |
| Almond 9 | 7.70 |
| Almond 10 | 8.04 |
| Coconut 1 | 6.41 |
| Coconut 2 | 6.40 |
| Coconut 3 | 5.90 |
| Coconut 4 | 5.87 |
| Coconut 5 | 5.98 |
| Coconut 6 | 6.38 |
| Coconut 7 | 6.33 |
| Coconut 8 | 6.31 |
| Coconut 9 | 5.91 |
| Coconut 10 | 5.93 |
| Cow 1 | 6.58 |
| Cow 2 | 6.7 |
| Cow 3 | 6.72 |
| Cow 4 | 6.78 |
| Cow 5 | 6.79 |
| Cow 6 | 6.71 |
| Cow 7 | 6.85 |
| Cow 8 | 6.80 |
| Cow 9 | 6.87 |
| Cow 10 | 6.80 |
| Plant hybrid | 7.43 |
| Oat 1 | 6.91 |
| Oat 2 | 7.32 |
| Oat 3 | 7.02 |
| Oat 4 | 7.04 |
| Oat 5 | 7.25 |
| Oat 6 | 7.21 |
| Oat 7 | 7.52 |
| Oat 8 | 7.68 |
| Oat 9 | 7.32 |
| Oat 10 | 6.60 |
| Rice 1 | 6.82 |
| Rice 2 | 6.82 |
| Rice 3 | 6.67 |
| soy 1 | 6.68 |
| soy 2 | 7.14 |
| soy 3 | 7.07 |
| soy 4 | 7.42 |
| soy 5 | 8.04 |
| soy 6 | 7.33 |
| soy 7 | 7.45 |
| soy 8 | 7.02 |
| soy 9 | 7.66 |
| soy 10 | 7.45 |

**Supplementary Table 8.** Random Forest Regression Hyperparameters and Tested Values. All combinations of the listed hyperparameters were systematically evaluated during model optimisation to identify the configuration yielding optimal predictive accuracy.

| **Hyperparameter** | **Tested Values** |
| --- | --- |
| n_estimators | 100, 200, 500 |
| max_depth | None, 10, 20 |
| min_samples_split | 2, 5, 10 |
| min_samples_leaf | 1, 2, 4 |
| max_features | 'auto', 'sqrt' |

**Supplementary Table 9.** Gradient Boosting Regression Hyperparameters and Tested Values. All combinations of the listed hyperparameters were systematically evaluated during model optimisation to identify the configuration yielding optimal predictive accuracy.

| **Hyperparameter** | **Tested values** |
| --- | --- |
| n_estimators | 100, 300, 500 |
| learning_rate | 0.01, 0.05, 0.1 |
| max_depth | 3, 5, 7 |
| min_samples_split | 2, 5 |
| min_samples_leaf | 1, 2 |

**Supplementary Table 10**. Wilcoxon signed-rank test for Random Forest and Gradient Boosting Regression models

| **Metric** | **RFR median** | **GBR median** | **W statistic** | **p-value** | **Interpretation** |
| --- | --- | --- | --- | --- | --- |
| RMSE | 1.14 | 1.32 | 5.00 | 0.63 | Not significant |
| R² | 0.82 | 0.77 | 1.00 | 0.13 | Not significant |

Model comparisons were performed using a paired Wilcoxon signed-rank test on fold-wise RMSE values from five identical cross-validation splits.

**Supplementary Table 11: Volatile compounds identified in the Formulation 1, Formulation 2, Ultra High temperature (UHT) cow and pasteurized cow milk types**

| **Compounds** | **Aroma descriptor** | **Estimated quantities in milk types (mg/L in internal standard equivalent)** | | | | |
| --- | --- | --- | --- | --- | --- | --- |
|  |  | **Formulation 1** | **Formulation 2** | **Pasteurized cow** | | **UHT cow** |
| 1-butanol | Sweet^4^ | 0.09 ± 0.01b | 0.08 ± 0.01b | | 0.31 ± 0.03a | ND |
| 3-methyl-1-butanol | Banana, floral, fruity, malt, wheat^3^ | ND | ND | | ND | 0.18 ± 0.03 a |
| 2-ethyl -1-hexanol | Oily^4^,  Sweet, floral^7^ | 0.33 ± 0.32a | ND | | ND | ND |
| 1-octanol | Bitter almond, fatty, green, rose^3^ | 0.30 ± 0.05b | 0.38 ± 0.06a | | 0.15 ± 0.04c | ND |
| 1-octen-3-ol | Mushroom^5^ | ND | 1.00 ± 0.13a | | ND | ND |
| 1-pentanol | Fruity, green, grain, mushroom, vanilla^3^ | 2.85 ± 0.13a | 2.48 ± 0.27b | | 0.15 ± 0.02c | 0.24 ± 0.08c |
| 2-butanone | Plastic ^5^ | ND | ND | | 0.37 ± 0.04b | 2.63 ± 0.46a |
| Furfuryl alcohol | Sweet^4^ | 0.08 ± 00b | 0.29 ± 0.04a | | ND | ND |
| 2-heptanone | Cooked ^5^,  Waxy, green^6^ | 0.40 ± 0.03b | 0.25 ± 0.06bc | | ND | 2.41 ± 0.40a |
| 2-heptenal (Z) | Pungent^4^ | 0.87 ± 0.21a | 0.37 ± 0.22b | | ND | ND |
| 2-methybutanal | Cooked, malty^5^ | ND | ND | | ND | 0.11 ± 0.01a |
| 2-nonanone | Sweet^4^ | ND | ND | | ND | 0.34 ± 0.07a |
| 2-octanone | Fatty, creamy^4^ | 0.12 ± 0.02a | 0.09 ± 0.04a | | ND | ND |
| 2-octenal (E) | Green, fatty^7^ | ND | 0.62 ± 0.36a | | ND | ND |
| 2-pentanone | Sweet, fruity^6^ | ND | ND | | ND | 2.98 ± 0.37a |
| 3-methylbutanal | Cooked, malty^5^ | ND | ND | | ND | 2.05 ± 0.22a |
| Pentanal | Grass, green ^8^ | 1.54 ± 0.08a | 0.85 ± 0.08b | | ND | ND |
| 3-octen-2-one | Mushroom ^2^ | 0.40 ± 0.03a | 0.26 ± 0.04 b | | ND | ND |
| Benzaldehyde | Cooked, nutty^5^ | ND | ND | | ND | 2.67 ± 0.38a |
| Butanoic acid | Acidic, cheesy^5^ | 0.66 ± 0.18 b | 0.08 ± 0.01c | | 2.07 ± 0.52a | ND |
| Delta- decalactone | Milky^9^ | ND | ND | | 0.15 ± 0.02b | 1.33 ± 0.15a |
| Delta-octalactone | Milky^9^ | ND | ND | | ND | 0.08 ± 0.02a |
| Dimethyl sulfone | Sulfur, burnt ^4^ | ND | ND | | 0.28 ± 0.01a | ND |
| Dimethyl sulfide | Sulfur ^5^ | ND | ND | | ND | 0.10 ± 0.02a |
| 2-pentyl-furan | Fruity, green^1^,  Caramel^2^ | 1.57 ± 0.10b | 0.57 ± 0.81c | | 0.09 ± 0.02c | 2.92 ± 0.38a |
| Heptanal | Earthy, fatty ^5^ | 0.39 ± 0.01a | 0.25 ± 0.04b | | 0.23 ± 0.01b | ND |
| Hexanal | Grassy, green ^7^ | 9.08 ± 0.83a | 5.48 ± 0.83b | | 0.38 ± 0.12c | ND |
| Maltol | Sweet ^4^ | 1.11 ± 0.09a | 0.61 ± 0.03b | | ND | ND |
| Nonanal | Rose-orange ^1^ | ND | ND | | ND | 0.29 ± 0.05a |
| Nonanoic acid | Cheese, dairy^4^ | ND | ND | | 0.26 ± 0.05a | ND |
| Octanal | Fruity, fatty ^8^ | 0.34 ± 0.04a | 0.28 ± 0.06a | | 0.29 ± 0.12a | 0.03 ± 0.01 b |
| Octanoic acid | Waxy, acidic^5^ | ND | ND | | 0.65 ± 0.30 a | ND |
| 2,5-dimethyl-pyrazine | Nutty, chocolate ^3^ | ND | ND | | ND | 0.69 ± 0.16a |
| Vanillin | Vanilla^7^ | 0.83 ± 0.01a | 0.62 ± 0.07b | | ND | 0.07 ± 0.02c |

Aroma descriptors were obtained from ^1^ (McCarron et al. 2024), ^2^(Li and Wang 2016), ^3^ (Pointke et al. 2022), ^4^(Jiang et al. 2024), ^5^(Jo et al. 2018), ^6^(Lan et al. 2025), ^7^(Vaikma et al. 2021), ^8^ (Feng et al. 2023), ^9^(Schütt and Schieberle 2017). The alcohols, aldehydes, ketones, pyrazines were semi-quantified using d13-hexanol, d12-hexanal, 2-hexanone, d6-2-methylpyrazine respectively. Furans, lactones and terpenes were semi-quantified using d_11_- 3-Methylbutanol.

**Supplementary Table 12: Analysis of Variance for assessor, presentation replicate, and sample as well as two-way interactions. F-ratios, probability level, degrees of freedom and mean square error (MSE)**

| **Attribute** | **Assessor** | **Presentation Replicate** | **Sample** | **Assessor× Presentation Replicate** | **Assessor × Sample** | **Sample × Presentation Replicate** | **MSE** |
| --- | --- | --- | --- | --- | --- | --- | --- |
| AP_White colour intensity | 47.59*** | 4.43* | 136.30*** | 0.93 | 3.16*** | 1.16 | 57.5 |
| AP_Yellow colour intensity | 37.35*** | 1.35 | 80.25*** | 1.31 | 4.67*** | 0.12 | 22.95 |
| AP_ Brown colour intensity | 21.95*** | 0.11 | 83.41*** | 0.72 | 2.27*** | 1.32 | 57.08 |
| A_Overall aroma intensity | 44.53*** | 0.62 | 37.06*** | 1.6 | 4.20*** | 0.34 | 44.46 |
| A_Cereal | 8.29*** | 0.09 | 21.64*** | 0.25 | 5.26*** | 0.8 | 67.1 |
| A_Beany | 6.21*** | 0.54 | 45.23*** | 0.6 | 2.90*** | 0.57 | 107.02 |
| A_Dairy | 11.30*** | 2.57 | 150.29*** | 1.77 | 3.28*** | 0.51 | 67.38 |
| A_Nutty | 7.69*** | 0.01 | 48.15*** | 2.02 | 4.52*** | 1.08 | 77.99 |
| A_Vanilla | 63.31*** | 0.31 | 17.47*** | 4.30*** | 5.03*** | 4.02** | 31.65 |
| A_Caramel | 18.29*** | 5.35* | 19.74*** | 2.36* | 1.92** | 0.46 | 54.73 |
| A_Coconut | 10.70*** | 0 | 691.04*** | 0.71 | 3.20*** | 0.42 | 17.57 |
| A_Chocolate | 9.04*** | 16.72*** | 173.11*** | 2.21* | 4.10*** | 1.88 | 28.83 |
| T_Sweetness | 22.39*** | 3.36 | 28.38*** | 0.73 | 2.06** | 0.31 | 115.36 |
| T_Sourness | 26.44*** | 0.66 | 2.12 | 2.73** | 2.23*** | 1.49 | 26.63 |
| T_Umami | 28.22*** | 1.45 | 5.62*** | 1.81 | 1.92** | 0.57 | 60.73 |
| T_Saltiness | 33.54*** | 0.18 | 2.23* | 1.34 | 1.93** | 0.74 | 23.68 |
| T_Bitterness | 37.70*** | 0.01 | 20.23*** | 0.6 | 6.06*** | 2 | 13.38 |
| FL_Overall flavour intensity | 14.55*** | 8.92** | 17.06*** | 0.77 | 5.31*** | 1.4 | 49.23 |
| FL_Beany | 19.85*** | 3.84 | 58.92*** | 0.89 | 4.23*** | 1.6 | 68.88 |
| FL_Chocolate | 15.47*** | 7.64** | 120.16*** | 3.67*** | 3.90*** | 0.99 | 27.32 |
| FL_Nutty | 9.75*** | 0.13 | 22.58*** | 1.25 | 3.82*** | 0.58 | 90.67 |
| FL_Metallic | 33.47*** | 0.87 | 8.94*** | 0.46 | 5.53*** | 1.1 | 22.12 |
| FL_Coconut | 15.67*** | 1.21 | 1475.75*** | 1.06 | 6.95*** | 1.61 | 8.58 |
| MF_Grittiness | 18.73*** | 0.29 | 21.64*** | 1.03 | 1.88** | 1.27 | 47.45 |
| MF_Creaminess | 15.93*** | 0.01 | 2.55* | 0.92 | 2.19** | 0.65 | 122.85 |
| MF_Astringency/dryness | 69.87*** | 7.67** | 4.70*** | 2.30* | 2.38*** | 0.89 | 51.6 |
| AT_Metallic | 39.27*** | 0.5 | 11.28*** | 1.43 | 4.96*** | 1.34 | 30.6 |
| AT_Coconut | 14.59*** | 0.42 | 550.30*** | 1.17 | 6.88*** | 0.62 | 14.84 |

*, P < 0.05; **, P < 0.01; ***, P < 0.001. A, aroma; FL, Flavor; AP, appearance; MF, mouthfeel. AT_Aftertaste

**Supplementary Figure 1.** Performance assessment of the formulation for prediction models Gradient Boosting Regression (GBR) and Random Forest Regression (RFR). Performance is based on R² (coefficient of determination) and RMSE (Root Mean Square Error) metrics across five independent runs. Values are reported as the mean ± standard deviation of four replicates (n = 5), with error bars representing standard deviation.

**References**

Feng, Xiaoxiao, Yiwen Zhu, and Yufei Hua. 2023. "New insights into the off-flavor improvement of soymilk by three grinding processing: Dry-blanching grinding, wet-blanching grinding, and wet-anaerobic grinding." *Food Chemistry: X* 20: 100892. <https://doi.org/https://doi.org/10.1016/j.fochx.2023.100892>.

Jiang, Kexin, Aolin Yang, Zheting Zhang, Kunli Xu, Huiyu Kuang, Fanyu Meng, and Bei Wang. 2024. "Identification of aroma-active compounds in milk by 2-dimensional gas chromatography-olfactometry-time-of-flight mass spectrometry combined with check-all-that-apply questions." *Journal of Dairy Science* 107 (11): 9124-9134. <https://doi.org/10.3168/jds.2024-24813>.

Jo, Y., D. M. Benoist, D. M. Barbano, and M. A. Drake. 2018. "Flavor and flavor chemistry differences among milks processed by high-temperature, short-time pasteurization or ultra-pasteurization." *Journal of Dairy Science* 101 (5): 3812-3828. <https://doi.org/https://doi.org/10.3168/jds.2017-14071>.

Lan, L., W. Wang, Y. Su, H. Xu, J. Han, X. Chi, Y. Xi, B. Sun, and N. Ai. 2025. "Exploration of milk flavor: From the perspective of raw milk, pasteurized milk, and UHT milk." *Food Chem X* 25: 102083. <https://doi.org/10.1016/j.fochx.2024.102083>.

Li, Y. H., and W. J. Wang. 2016. "Short communication: Formation of oxidized flavor compounds in concentrated milk and distillate during milk concentration." *Journal of Dairy Science* 99 (12): 9647-9651. <https://doi.org/https://doi.org/10.3168/jds.2016-11619>.

McCarron, R., L. Methven, S. Grahl, R. Elliott, and S. Lignou. 2024. "Oat-based milk alternatives: the influence of physical and chemical properties on the sensory profile." *Front Nutr* 11: 1345371. <https://doi.org/10.3389/fnut.2024.1345371>.

Pointke, Marcel, Elke H. Albrecht, Katrin Geburt, Martina Gerken, Imke Traulsen, and Elke Pawelzik. 2022. "A Comparative Analysis of Plant-Based Milk Alternatives Part 1: Composition, Sensory, and Nutritional Value." *Sustainability* 14 (13). <https://doi.org/10.3390/su14137996>.

Schütt, Jessica, and Peter Schieberle. 2017. "Quantitation of Nine Lactones in Dairy Cream by Stable Isotope Dilution Assays Based on Novel Syntheses of Carbon-13-Labeled γ-Lactones and Deuterium-Labeled δ-Lactones in Combination with Comprehensive Two-Dimensional Gas Chromatography with Time-of-Flight Mass Spectrometry." *Journal of Agricultural and Food Chemistry* 65 (48): 10534-10541. <https://doi.org/10.1021/acs.jafc.7b04407>.

Vaikma, Helen, Aleksei Kaleda, Julia Rosend, and Sirli Rosenvald. 2021. "Market mapping of plant-based milk alternatives by using sensory (RATA) and GC analysis." *Future Foods* 4: 100049. <https://doi.org/https://doi.org/10.1016/j.fufo.2021.100049>.
